# Aging alters the immunological response to ischemic stroke

**DOI:** 10.1007/s00401-018-1859-2

**Published:** 2018-05-11

**Authors:** Rodney M. Ritzel, Yun-Ju Lai, Joshua D. Crapser, Anita R. Patel, Anna Schrecengost, Jeremy M. Grenier, Nickolas S. Mancini, Anthony Patrizz, Evan R. Jellison, Diego Morales-Scheihing, Venugopal R. Venna, Julia K. Kofler, Fudong Liu, Rajkumar Verma, Louise D. McCullough

**Affiliations:** 1 Department of Anesthesiology, Center for Shock, Trauma, and Anesthesiology Research, University of Maryland School of Medicine, Baltimore, MD 21201 USA; 2 Department of Neuroscience, University of Connecticut Health Center, Farmington, CT 06030 USA; 3 Cizik School of Nursing, University of Texas Health Science Center at Houston, Houston, TX 77030 USA; 4 Immunology Department, University of Connecticut Health Center, Farmington, CT 06030 USA; 5 Department of Neurology, McGovern Medical School, University of Texas Health Science Center at Houston, 6431 Fannin Street, Houston, TX 77370 USA; 6 Department of Pathology, University of Pittsburgh School of Medicine, Pittsburgh, PA 15261 USA

**Keywords:** Aging, Bone marrow, Rejuvenation, Inflammation, Stroke, Neutrophils

## Abstract

The peripheral immune system plays a critical role in aging and in the response to brain injury. Emerging data suggest inflammatory responses are exacerbated in older animals following ischemic stroke; however, our understanding of these age-related changes is poor. In this work, we demonstrate marked differences in the composition of circulating and infiltrating leukocytes recruited to the ischemic brain of old male mice after stroke compared to young male mice. Blood neutrophilia and neutrophil invasion into the brain were increased in aged animals. Relative to infiltrating monocyte populations, brain-invading neutrophils had reduced phagocytic potential, and produced higher levels of reactive oxygen species and extracellular matrix-degrading enzymes (i.e., MMP-9), which were further exacerbated with age. Hemorrhagic transformation was more pronounced in aged versus young mice relative to infarct size. High numbers of myeloperoxidase-positive neutrophils were found in postmortem human brain samples of old (> 71 years) acute ischemic stroke subjects compared to non-ischemic controls. Many of these neutrophils were found in the brain parenchyma. A large proportion of these neutrophils expressed MMP-9 and positively correlated with hemorrhage and hyperemia. MMP-9 expression and hemorrhagic transformation after stroke increased with age. These changes in the myeloid response to stroke with age led us to hypothesize that the bone marrow response to stroke is altered with age, which could be important for the development of effective therapies targeting the immune response. We generated heterochronic bone marrow chimeras as a tool to determine the contribution of peripheral immune senescence to age- and stroke-induced inflammation. Old hosts that received young bone marrow (i.e., Young → Old) had attenuation of age-related reductions in bFGF and VEGF and showed improved locomotor activity and gait dynamics compared to isochronic (Old → Old) controls. Microglia in young heterochronic mice (Old → Young) developed a senescent-like phenotype. After stroke, aged animals reconstituted with young marrow had reduced behavioral deficits compared to isochronic controls, and had significantly fewer brain-infiltrating neutrophils. Increased rates of hemorrhagic transformation were seen in young mice reconstituted with aged bone marrow. This work suggests that age alters the immunological response to stroke, and that this can be reversed by manipulation of the peripheral immune cells in the bone marrow.

**Electronic supplementary material:**

The online version of this article (10.1007/s00401-018-1859-2) contains supplementary material, which is available to authorized users.

## Introduction

Stroke is the fourth leading cause of death and the primary cause of long-term disability worldwide, and remains a major economic and healthcare burden [55]. Over 80% of ischemic strokes occur in people aged 65 years and older. The elderly have increased stroke prevalence, greater stroke-related mortality and disability, and are at higher risk of complications related to thrombolytic treatment compared to younger patients [6, 69, 71]. The increased vulnerability of the elderly to stroke is associated with distinct, non-modifiable risk factor profiles and mechanisms of injury compared to younger patients [11, 35]. Although the etiology of stroke differs in older age groups [60], there is a relative paucity of studies comparing the response to ischemia in different age groups in both humans [39, 66] and rodents [8, 51, 53, 95]. Given that inflammation is critical driver of stroke recovery, and that aging adversely impacts the immune system, the role of the systemic immune response in older stroke patients remains a fundamental unanswered question. In this work we first evaluated the effects of aging on the immune response to stroke and then replaced the peripheral bone marrow to determine the response to injury in the aged brain.

Normal aging can have profound effects on innate immune function and its response to injury, yet surprisingly few studies have investigated the role that aging has on the peripheral immune response to brain injuries such as stroke. Although reports on age-related infarct changes have been mixed [19, 61, 77], growing evidence points to key elements of the systemic inflammatory response as a possible explanation for the higher mortality rates, increased susceptibility to secondary infection, and poor behavioral recovery seen in the elderly [14, 19, 53]. Our laboratory and others have repeatedly shown that aged mice have smaller infarct sizes compared to their younger counterparts, despite having disproportionately worse outcomes [52]. Indeed, recent data suggest that systemic inflammation can compromise survival and blood–brain barrier integrity independently of infarct size [15]. And while some studies show aged mice have quantitatively fewer numbers of brain-infiltrating leukocytes after stroke, likely a result of having smaller infarcts, the composition and qualitative response of those leukocytes to injury remain unexplored. Previous findings imply that aging may alter the immunological response to ischemic brain injury, characterized by higher CCL5/RANTES concentrations and increased neutrophil chemotaxis [19]; however, the extent that these age-related changes alter stroke outcomes is unknown.

Previous work has shown that circulating factors from young mice given to old recipients can reverse age-related deficits in cognition. The origin of these rejuvenating factors is unknown [7, 9]. Given the importance of the bone marrow response to ischemic brain injury, we generated heterochronic bone marrow chimeras using young and aged hosts to manipulate the peripheral immune response to determine if immune cells, and the factors they secrete, have the potential to influence behavioral and motor function at baseline and after ischemic brain injury. Aging skewed the bone marrow response to stroke, resulting in the biased recruitment of pro-inflammatory neutrophils, increased hemorrhagic transformation, and poorer acute behavioral outcomes. This phenotype was recapitulated in young hosts after transplants of aged bone marrow. Aged animals repopulated with young bone marrow had better behavioral performance even in the absence of injury and had significantly improved outcomes after ischemic stroke. Importantly, infarct damage was not altered by bone marrow transplants, indicating that acute behavioral deficits may be largely driven by the peripheral immune response. We conclude that the neuroinflammatory response to stroke is a major driver of post-stroke recovery.

## Materials and methods

### Mice/animals

Young (12–14 weeks; 24.5 ± 0.8 g) and aged (18–20 months; 33.1 ± 1.5 g) adult male mice from Charles River Laboratories (Wilmington, MA) pair-housed on sawdust bedding in a pathogen-free facility (light cycle 12/12 h light/dark) were maintained in house until use. All animals had access to chow and water ad libitum. All studies were conducted in accordance with the United States Public Health Service’s Policy on Human Care and Use of Laboratory Animals, and all procedures were performed in accordance with NIH guidelines for the care and use of laboratory animals and approved by the Institutional Animal Care and Use Committee of the University of Connecticut Health Center and the McGovern Medical School.

### Ischemic stroke model

Cerebral ischemia was induced by reversible middle cerebral artery occlusion (MCAO, 25–32 g mice) under isoflurane anesthesia as previously described [47]. Young and aged animals were subjected to 60 min of MCAO followed by 72 h of reperfusion. Rectal temperatures were maintained at approximately 37 °C during surgery and ischemia with an automated temperature control feedback system. To achieve equivalent occlusion (i.e., blood flow reduction), 0.21-mm and 0.23-mm silicone-coated sutures were utilized in young and aged mice, respectively, as previously validated [52]. Cerebral blood flow flux demonstrated similar reductions between young (84.3% ± 6.8) and aged (86.6% ± 5.2) mice, and returned to 90% of baseline in both groups within 30 min of reperfusion, consistent with prior work [51]. Sham-operated animals underwent the same surgical procedure, but the suture was not advanced into the internal carotid artery. All mice were selected for sham or stroke (MCAO) surgery in a randomized manner and all analyses were performed blinded to surgical conditions.

### Clinical assessment

Body weight was monitored 1 day before and every day after ischemia. Rectal temperatures were recorded during ischemia (immediately after removing from a feedback-controlled surgical heating pad) and immediately prior to killing. Neurological deficits were assessed by Bederson-score from 0 (no deficit) to 4 (severe deficit) with minor modifications [5].

### Behavioral testing

Animals were tested on each behavioral task twice, 3 days prior to surgery to establish a baseline reference and again on the day of killing. Behavioral data obtained from bone marrow chimera experiments are representative of two biological replicates (i.e., from different litters) for each group. The testing was done at a fixed time in the morning (09:00–11:00) by investigators blinded to the donor status of each group. All behavioral testing equipment and surfaces were cleaned with 70% ethanol before and after testing for each animal. Mice were tested in the following order: neurological scoring, open field, rotarod, wirehang, and tail suspension.

### Hanging wire

The hanging wire tests both limb strength and balance after stroke. This test was performed as described by [31], with slight modification. A wire cage top (18″ × 9″) was used for this experiment. The mouse was placed on the center of the wire lid and the lid was slowly inverted and placed 12″ above the cage. Latency to fall from the wire was recorded. The average time of three trials was taken. Mice with a latency of less than 3 s were excluded from the study, whereas a maximum cutoff of 120 s was assigned this value.

### Open field

The open field measures spontaneous locomotor activity in a novel environment [94]. The mouse was placed in the open-field chamber (15′′ × 15′′) in a dark room. Locomotor activity was quantified as the total number of beam breaks by a computer-operated PAS Open-Field system (San Diego Instruments, San Diego, CA). Anxiety levels were measured by taking the percentage of the total beam breaks in center of the chamber versus the perimeter [62]. Each testing session was 20 min long and the data were collected at 60-s intervals.

### Rotarod

Mice were placed on a rotating cylindrical rod accelerating from 2 rotations per minute (rpm) to 20 rpm, over a span of 5 min [86]. Non-injured and stroke subjects were given 5 trials on the rotarod on day − 3 and + 3 of testing. A 5-min break was given between the trials. The latency of the subject to fall from the rotating rod was recorded for each trial (in seconds), and the average latency was used for further analysis.

### Tail suspension

The tail suspension test (TST) was performed as described previously [90] with minor modifications. Briefly, the mice were individually suspended from the tail suspension apparatus, 60 cm above the surface of the table. The experiment was recorded for 6 min using a digital video camera (JVC Everio, Victor Company, Japan). A trained observer that was blinded to the treatment group recorded the duration of immobility.

### Gait dynamics

Mouse gait dynamics were obtained using a motorized DigiGait^®^ treadmill (with a transparent belt and digital video camera mounted underneath) by ventral plane videography and analyzed with DigiGait^®^ software (Mouse Specifics, Inc. Quincy, MA). One day prior to behavioral testing, mice were acclimated to the testing room for 30 min. Gait analysis was performed on a flat platform at a speed of 12 cm/s, with an average of 5 s of video analyzed for each mouse. Subjects were given three trials, with 5-min rest periods. In between animals, the treadmill was wiped down with 70% ethanol. Right and left paw data were averaged for the fore paws of each animal, and data for each animal in a group were averaged.

### Terminal histopathology

All animals were euthanized at 72 h after stroke with an avertin overdose (i.p). A separate cohort of animals was euthanized for histological analyses to assess infarct volume. Transcardial perfusion was performed with cold PBS followed by 4% paraformaldehyde; the brain was post-fixed for 24 h and placed in cyroprotectant (30% sucrose). The brains were cut into 40-μm-thick free-floating sections on a freezing microtome and every eighth slice was stained by cresyl violet staining to evaluate ischemic cell damage. The images were digitalized and infarct volume was analyzed by a blinded investigator using computer software (Sigma scan Pro5) as previously described [47].

### Human brain tissue

All human tissue samples were obtained from the University of Pittsburgh neurodegenerative brain bank with appropriate ethics committee approval (Committee for Oversight of Research and Clinical Training Involving Decedents). All cases meeting inclusion criteria in a database search of the University of Pittsburgh brain bank and which had sufficient brain tissue available were included in the study. The database search was performed by the brain bank manager who was not involved in any subsequent analysis. All slides were analyzed by an investigator blinded to case demographics and age of stroke. Representative brain tissue samples were fixed in formalin and embedded in paraffin. Since information of clinical stroke onset to time of death was unavailable for some cases, stroke age was determined by histopathologic criteria. Acute infarcts were characterized by neuropil vacuolization and acute ischemic neuronal injury with cytoplasmic eosinophilia and nuclear pyknosis. The appearance of foamy macrophages and indicated the transition from acute to subacute stages. Subacute infarcts further showed endothelial proliferation and neovascularization, the development of reactive astrocytosis and, over time, fading of necrotic ischemic neurons.

### Immunohistochemistry

Human brain sections were deparaffined in xylene, rehydrated through a graded series of ethanol baths. The sections were then placed into sodium citrate buffer (pH 6.0) and heated in a microwave oven for 10 min. After cooling, the sections were washed with PBS for 5 min twice, and then treated with 3% H_2_O_2_ for 10 min at room temperature (RT) to quench endogenous peroxidase and washed three times with PBS. Non-specific antibody binding was blocked by incubation of the sections with 3% BSA in PBS for 30 min at RT. After incubating with primary antibodies [anti-MPO (ab9535) 1:50, MMP-9 (SMC-396D) 1:250, laminin alpha 5 (ab210957) 1:100, and claudin-5 (34-1600) 1:100] overnight at 4 °C, the sections were washed with PBS for 5 min three times. Subsequently, an anti-rabbit or mouse-alkaline phosphatase (AP) secondary antibody was applied for 30 min at RT, and washed with PBS for 5 min three times. Finally, the color reaction was started by adding AP substrate for 5–10 min and stopped by washing the slides in PBS. All slides were counterstained with hematoxylin. Images were collected using a Leica microscope with a Leica DFC 310 FX digital camera (Leica, Heerbrugg, Switzerland). To quantify levels of expression of MPO and MMP-9, hemorrhage, and hyperemia, the sections were examined with the × 4 objective. Each section was divided into nine fields. The stained area was divided by the total area in each field. The percentage of stained area was counted in nine fields in each section to obtain the percentage of stained cells by an investigator blinded to sample age. Pearson or Spearman correlation analyses were conducted to compare the results of a total 30 brain samples, including 15 controls (37–95 years old) and 15 ischemic stroke subjects (44–89 years old). Of these, eight were confirmed to have no treatment with lytic therapy for their strokes, and four other samples were collected before 1998, when thrombolytics were rarely used. Samples were also classified based on pathological evidence of cerebral amyloid angiopathy into none, mild, moderate, or severe.

### Hemorrhagic transformation

Brain slices were taken at the same distance from bregma (0.5 mm anterior to bregma) and three sections/animal were analyzed in the area of the infarct. Hemorrhagic transformation was calculated based on the area of oxidized hemoglobin present in the ischemic hemisphere. Using Image J software (National Institutes of Health, Maryland, USA), each image was adjusted to grayscale and the threshold adjusted such that only the areas of oxidized hemoglobin showed signal. This area was divided by the area of the ipsilateral hemisphere and multiplied by 100 to obtain the percent of the hemisphere containing oxidized hemoglobin resulting from hemorrhagic transformation.

### Enzyme-linked immunoabsorbent assay (ELISA)

Plasma and brain cytokine concentrations were determined by ELISA (Bio-Plex Pro Mouse Cytokine Assay, Bio-Rad Laboratories). In brief, mice were euthanized by avertin injection and blood was collected by cardiac puncture into heparin-coated syringes. Samples were centrifuged (13,000×*g* for 10 min at 4 °C) and plasma was collected and stored frozen (− 80 °C) until assay. Brain hemispheres were collected and homogenized in ice-cold lysis buffer containing a protease inhibitor cocktail (Roche Diagnostics). Homogenates were processed as previously described [65]. Briefly, 25 µl of plasma and 150 µg of whole cell lysate brain protein were loaded into each well in duplicate. Samples were assayed according to the manufacturer’s instructions using a Luminex 200 (Luminex Corporation, Austin, TX, USA) magnetic bead array platform. MMP-9 concentrations in the plasma and brain (200 µg/well) were obtained by ELISA assay (Cat # LS-F5604, LifeSpan BioSciences, Seattle, WA) in young and aged mice as per the manufacturer’s instructions.

### Hemoglobin measurements

Prior to dissection, mice were transcardially perfused with PBS containing 0.16 mg/mL of heparin to remove extraneous red blood cells. Ipsilateral sham and ischemic hemispheres were harvested and immediately placed into 15-mL tubes containing 2 mL of PBS. The tissue was homogenized and centrifuged at 10,000×*g* for 15 min at 4 °C. The supernatant was plated in duplicate according to the manufacturer’s instructions for the Hemoglobin Colorimetric Assay kit (Caymen Chemical Company, Ann Arbor, MI).

### Tissue processing for flow cytometry

Mice were euthanized, transcardially perfused with 60 mL cold, sterile PBS, and the brains were harvested. Blood was drawn by cardiac puncture with heparinized needles. Red blood cell lysis was achieved by three consecutive 10-min incubations with Tris–ammonium chloride (Stem Cell Technologies). Leukocytes were isolated from brain hemispheres as previously described [64]. Cells were washed and blocked with mouse Fc Block (CD16/CD32, eBioscience) prior to staining with primary antibody-conjugated fluorophores: CD45-eF450 (30-F11), CD11b-APCeF780 (M1/70), Ly6C-PerCP-Cy5.5 (HK1.4), Ly6G-PE or PE-Cy7 (1A8), SIRP-alpha-APC (P84), and CASE-AF350 viability dye (Invitrogen). All antibodies were commercially purchased from eBioscience. A gating strategy was designed as described in [64]. Resident microglia were identified as the CD45^int^ CD11b^+^Ly6C^−^ population, whereas bone marrow-derived leukocytes were identified as CD45^hi^. Neutrophils were identified as CD45^hi^CD11b^+^Ly6C^+^Ly6G^+^ and monocytes were identified as CD45^hi^CD11b^+^Ly6C^+^Ly6G^−^ cells. Cell counts obtained from the ischemic hemispheres of chimeric mice are representative of two biological replicates. Cell type-matched fluorescence minus one (FMO) controls were used to determine the positive gating for each antibody.

To study phagocytic activity, fluorescent latex beads (Fluoresbrite Yellow Green (YG) carboxylate microspheres; 1 µm diameter; Polysciences) were added to sorted leukocytes in a final dilution of 1:100 as described [93].

For detection of reactive oxygen species (ROS), microglial cells were incubated with redox-sensitive DHR (5 µM; Ex/Em: 495/520) fluorogenic cell-permeant dye (Life Technologies, Invitrogen). Cells were incubated for 20 min at 37 °C, washed three times with FACS buffer (without NaAz), and then stained for surface markers including CASE. After loading cells with DHR, each sample was separated into two tubes, one kept on ice and one at 37 °C.

For intracellular cytokine staining, an in vivo Brefeldin A protocol was followed as described previously [63]. Isolated cells were resuspended in Fc block, stained for surface antigens and washed in 100 µl of fixation/permeabilization solution (BD Biosciences) for 20 min. Cells were then washed twice in 300 µl permeabilization/wash buffer (BD Biosciences) and resuspended in an intracellular antibody cocktail containing TNF-PE-Cy7 (eBioscience, MP6-XT22) and MMP-9-PE (StressMarq Biosciences, S51-82), and then fixed.

### Bone marrow chimera generation

Wildtype male C57BL/6 young and aged mice were lethally irradiated (two doses of 5–6 Gy) in a Gammacell 40 research irradiator and 5 × 10^5^ nucleated GFP-expressing donor bone marrow cells (of 10 wks or 18 months of age) were injected retro-orbitally [72]. Chimeras were maintained on sulfamethoxazole/trimethoprim antibiotics in their drinking water 1 day prior and five wks following irradiation. Chimeras were allowed to reconstitute for 8 wks following transplantation and only those with > 85% GFP reconstitution (determined by FACS analysis of blood) were included in this study. Young and old mice were randomized immediately following irradiation, prior to injection of young or old donor cells.

### Statistical analyses

Data from individual experiments are presented as mean ± SD and assessed by Student’s *t* test (for comparison between two experimental groups) or one-way ANOVA with Tukey’s or Dunnett’s post hoc test for multiple comparisons (GraphPad Prism Software Inc, San Diego, CA, USA). For two-way ANOVA, significant differences between paired comparisons were conducted with the Holm–Sidak test, whereas group analysis was compared using Tukey’s post hoc correction. The neurological deficit scores, being ordinal in nature, were analyzed using the Mann–Whitney *U* test. Postmortem human brain samples were evaluated by Kruskal–Wallis test with Dunn’s correction for multiple comparisons. Pearson or Spearman correlation analysis was used to assess the relationship between age and histopathology. A power analysis based on preliminary data was performed on all groups to confirm appropriateness of sample size (G power). Significance was set at *p* ≤ 0.05.

## Results

### Old mice have significantly worse neutrophilia, hemorrhagic transformation, and neurological scores after stroke relative to infarct volume as compared to young mice

**Fig. 1:**
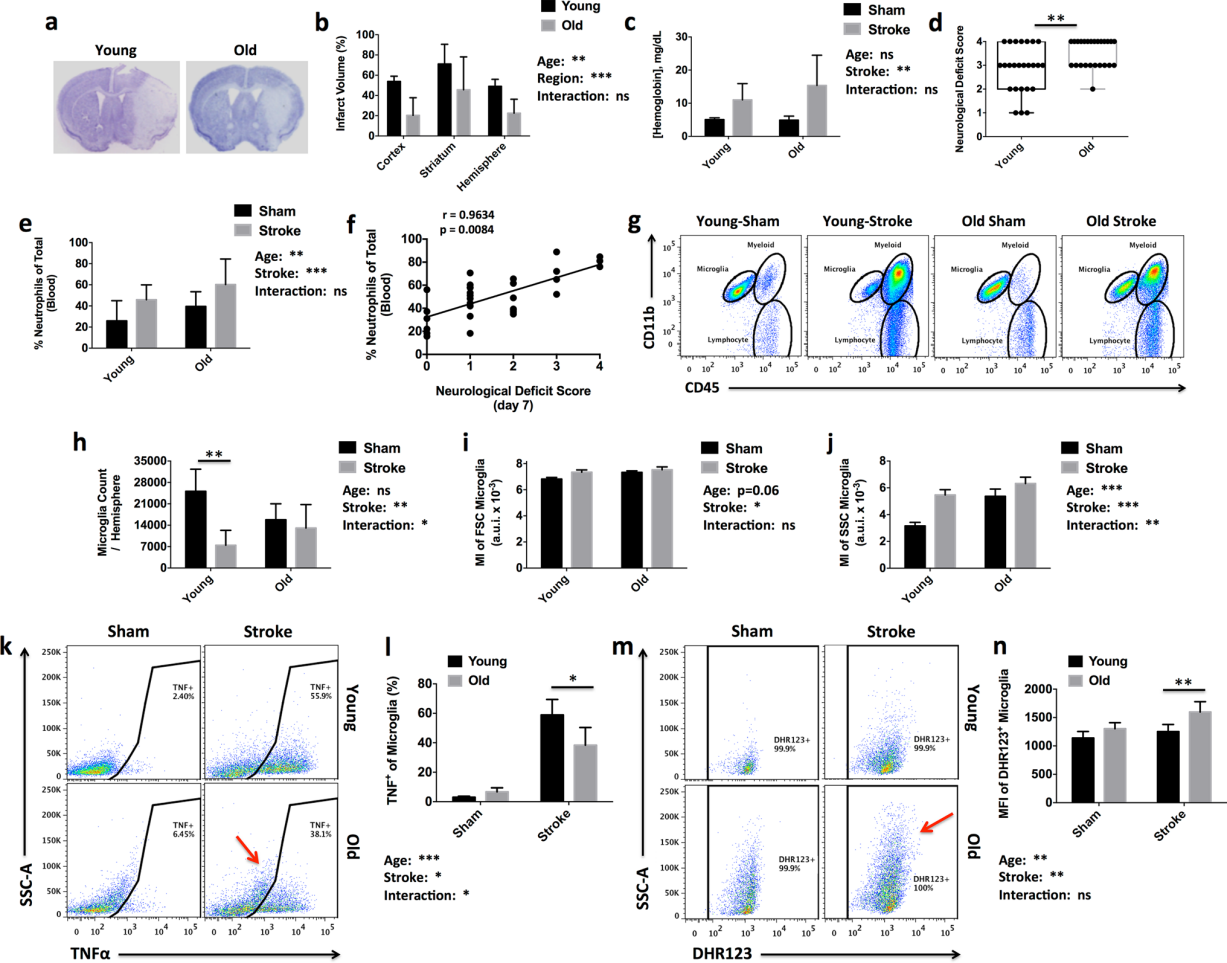
Age-related worsening of neurological deficits is associated with compositional changes in circulating and infiltrating myeloid cells in the post-ischemic brain. Representative coronal brain sections stained with cresyl violet show the extent of infarct volume in wildtype young and old C57B/6 J mice at 72 h after middle cerebral artery occlusion (**a**). The percentage of area infarcted was measured and quantified (**b**; *N* = 7/group). Hemorrhagic transformation was evaluated by measuring hemoglobin concentration in the ipsilateral hemisphere (**c**; *N *= 5 sham and 8–11 stroke/group). Neurological deficit scores were significantly higher in older mice after stroke compared to young mice (**d**; *N *= 25/group). The percentage of CD45^+^CD11b^+^Ly6C^+^Ly6G^+^ neutrophils (of total CD45^+^) in blood was quantified by flow cytometry (**e**). Spearman’s correlation analysis revealed a positive correlation between the severity of neutrophilia and the severity of neurological deficit score at 1 week after stroke in a separate cohort of young mice (**f**; *N *= 31). Representative dot plots illustrate the identification of CD45^int^CD11b^+^-resident microglia and CD45^hi^CD11b^+^-brain-infiltrating leukocytes in young and old mice at 72 h after stroke (**g**). Microglial (CD45^int^CD11b^+^Ly6C^-^) cell counts per ipsilateral hemisphere are shown (**h**; *N *= 4 sham and 7 stroke/group). Relative cell size, as measured by forward scatter intensity (**i**), and relative cell granularity, as measured by side scatter intensity (**j**), were quantified. Microglia activation was determined by assessing TNF production at 72 h. Representative dot plots show the relative level of microglial TNF expression in young and old mice after stroke and in sham controls (**k**). The red arrow highlights the failure of high-side scatter, aged microglia to elicit TNF production in response to ischemic stimuli. The percentages of TNF-positive microglia in the ischemic hemisphere were quantified (**l**; *N *= 4 sham and 6 stroke/group). Representative dot plots show the relative level of microglial ROS production as assessed by DHR123 in young and old mice after stroke and in sham controls (**m**). The red arrow highlights the elevated ROS production exhibited by high-side scatter, aged microglia after ischemic injury. The mean fluorescence intensities of DHR123-positive microglia in the ischemic hemisphere were quantified (**n**; *N *= 6 sham and 6–7 strokes/group). Error bars show mean SD. *DHR123* dihydrorhodamine 123, *TNF* tumor necrosis factor and *SD* standard deviation. These data are representative of two independent experimental cohorts. In **b**, **c**, **e**, **h**, **i**, **j**, **l**, and **n**, statistical analysis was performed by two-way ANOVA with a follow-up Tukey multiple-comparison test. Group effects are shown. In **d**, statistical analysis was performed using unpaired, nonparametric, Mann–Whitney’s *t* test, wherein each dot represents an individual mouse. *p < 0.05; **p < 0.01; ***p < 0.001

Our laboratory and others have previously demonstrated that old mice have less histological injury than their young adult counterparts at 24 h after stroke, despite having worse survival rates and long-term behavioral recovery [48, 52, 53]. We hypothesized that age-related changes in the inflammatory response to ischemia are responsible for this disparity. The effect of aging on stroke outcome was examined at 72 h, a critical time period that coincides with peak inflammation in the brain. The mortality rate for aged mice was higher after MCAO than for young (44%; 25/57 total mice vs. 20%; 9/45 total mice, respectively), consistent with previous reports [14, 95]. Despite a ~ 50% reduction in infarct volume (Fig. 1a, b), hemorrhagic transformation was increased in old mice (Fig. 1c) and neurological deficit scores were significantly worse (Fig. 1d). A significantly higher percentage of circulating neutrophils were found in the blood of aged mice after stroke compared to young mice, indicating age-related exacerbation of the bone marrow response (Fig. 1e). Neutrophilia was strongly associated with poor neurological outcome in aged mice after stroke at day 3; however, even in young mice at later time points (day 7) we observed a significant correlation between elevated blood neutrophil counts and more severe neurological deficit scores, indicating that stroke-induced bone marrow activation may aggravate neurological recovery (Fig. 1f).

### Microglial production of tumor necrosis factor (TNF) and reactive oxygen species (ROS) after ischemic stroke is altered with age

Because inflammation is a critical driver of ischemic injury and outcome, we next assessed the effect of aging on the neuroinflammatory response (Fig. 1g). Microglia counts were higher in young sham mice compared to old mice; however, after stroke the number of young microglia was significantly decreased, likely due to infarct severity (Fig. 1h). The physical characteristics of aged microglia, as measured by forward scatter and side scatter intensity, were profoundly different than young (Fig. 1i, j, respectively). In both age groups, microglial cell size and granularity were significantly increased after stroke. Interestingly, however, the size and complexity of young microglia after stroke were similar to that seen in aged sham microglia. To determine whether exacerbated microglia activation could account for poor outcomes in older mice, we assessed TNF protein production after stroke, which is known to correlate with poor outcome [44]. No age-related change in TNF expression was found in the infiltrating Ly6C + monocyte population (SF1). Although baseline production of TNF was modestly elevated in old microglia, young mice exhibited significantly higher microglial TNF expression after stroke, consistent with their larger infarct size (Fig. 1k, l). A subset of microglia found in aged mice (~ 15–20%) exhibited unusually high granularity, autofluorescence, and lipofuscin-like material. These previously described CD45^int^CD11b^+^Side Scatter^hi^ ‘dystrophic’ microglia had noticeably absent TNF production after stroke (SF2), suggesting these senescent-like populations may be refractory or have dysregulated responses to ischemic stimuli [65]. We also assessed microglial production of reactive oxygen species, which contribute to oxidative stress and neuronal injury [88]. An age-related increase in microglial ROS production was demonstrated after stroke (Fig. 1m, n). Interestingly, Side Scatter^hi^ microglia from old mice exhibited markedly higher ROS production after stroke relative to other microglia populations (SF2), suggesting an important role for age-associated dystrophic microglia in mediating neuroinflammation in older mice.

### Old mice have an altered composition of brain-infiltrating myeloid cells after stroke that exhibit age-related deficits in their functional response

**Fig. 2:**
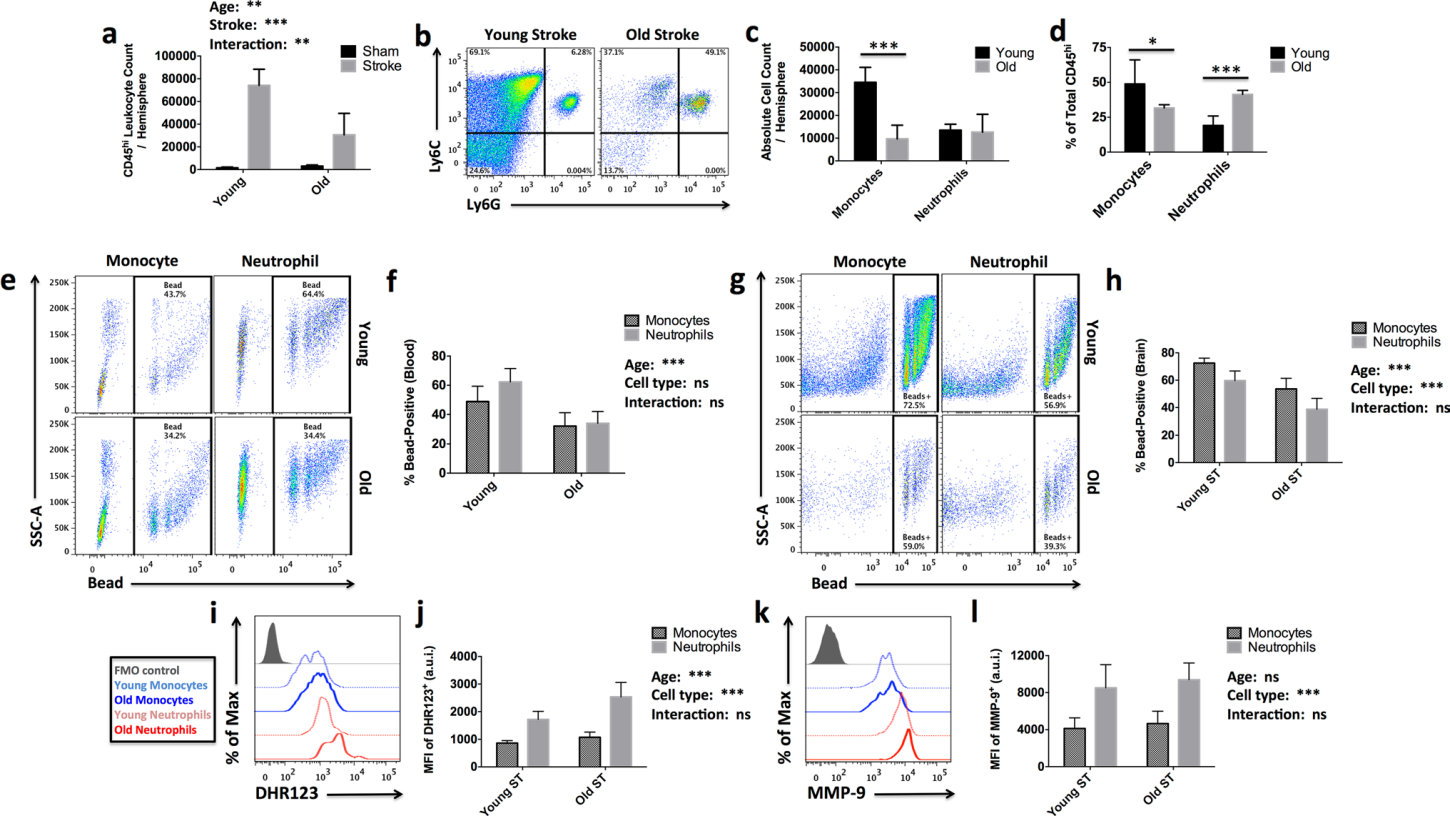
Age-related dysregulation of the leukocyte response in the ischemic brain. Absolute counts of brain-infiltrating CD45^hi^ leukocytes were quantified (**a**; *N *= 4 sham and 7 stroke/group). Representative dot plots illustrate the differential recruitment of myeloid cell (CD45^hi^CD11b^+^) subsets to the ischemic brain with age (**b**). Cell-specific FMO controls were used to determine positive gating. The absolute number of infiltrating monocytes (CD45^hi^CD11b^+^Ly6C^+^Ly6G^-^) and neutrophils (CD45^hi^CD11b^+^Ly6C^+^Ly6G^+^) in the young and aged ischemic brain is shown (**c**). The percent of brain-infiltrating monocytes versus neutrophils of total CD45^hi^ were altered with age after stroke (**d**). A representative dot plot depicts the percentage of phagocytic (bead-positive) monocytes (CD45^hi^CD11b^+^Ly6C^+^Ly6G^-^; blue) and neutrophils (CD45^hi^CD11b^+^Ly6C^+^Ly6G^+^; red) isolated from the blood of young (dotted) and old (solid) mice (**e**). Cell-specific FMO controls were used to determine positive gating (shade gray). The percentage of bead-positive circulating cells is shown (**f**; *N *= 5/group). The phagocytic activity of monocytes and neutrophils isolated from young and old ischemic hemispheres was examined (**g**) and quantified (**h**; *N* = 6–7/group). Cells were isolated from young and old ischemic hemispheres at 72 h and labeled with dihydrorhodamine 123 (DHR) to assess reactive oxygen species (ROS) production (**i**). The relative level of ROS production between young and old monocytes and neutrophils is shown (**j**; *N* = 6–7/group). A representative histogram illustrates the relative production of MMP-9 between monocytes and neutrophils isolated from the ischemic hemispheres of young and old mice at 72 h after stroke (**k**). The mean fluorescence intensity of MMP-9 expression is quantified (**l**; *N *= 5/group). Error bars show mean SD. *a.u.i.* arbitrary units of intensity, *CCL5* chemokine (C–C motif) ligand 5, *MFI* mean fluorescence intensity and *SD* standard deviation. In **a**, **c**, **d**, **f**, **h**, **j**, and **l**, statistical analysis was performed using two-way ANOVA with post hoc Tukey test for multiple comparisons. Group effects are shown. ^**#**^*p *< 0.05; *p < 0.05; **p < 0.01; ***p < 0.001

Stroke-induced activation of bone marrow myeloid progenitors via sympathetic innervation results in the robust production and recruitment of myeloid cells to the injured brain [17]. Age-related increases in plasma and brain concentrations of CCL5 (RANTES), a pro-inflammatory chemokine involved in leukocyte migration, were found acutely after stroke (SF3); this was only seen in aged mice. Similar increases in CCL2 expression were also noted (SF3). We found that leukocyte infiltration was significantly attenuated in older mice, correlating with infarct volumes (Fig. 2a). However, most importantly, the composition of the infiltrating leukocyte populations was significantly different, with monocytes making up a significantly larger proportion of the immune cells migrating into the young brain, and neutrophils representing the predominant immune cells infiltrating into the aged brain after stroke (Figs. 2b–d and SF4). Given the noted effects of aging on immune cell function and the recently defined roles for these two myeloid cells in brain injury, we hypothesized that differences in the bone marrow response to stroke could underlie age-related stroke severity.

### The effects of aging and ischemic stimuli on neutrophil responses are exacerbated in old mice

Age-related changes in monocyte and neutrophil function could influence post-stroke neurobehavioral deficits and hinder recovery. To assess this we performed a series of ex vivo functional experiments to determine the relative activation status of these cells in the ischemic brain. We found that the phagocytic potential of circulating blood monocytes and neutrophils is impaired with age (Fig. 2e, f). Neutrophils isolated from the (72 h) ischemic brain of old mice were significantly less efficient at phagocytosing beads compared to monocytes and neutrophils isolated from the ischemic brain of young mice (Fig. 2g, h). Compared to monocytes and young neutrophils, aged neutrophils exhibited significantly greater reactive oxygen species production in the ischemic brain (Fig. 2i, j). Similarly, MMP-9 production was twofold higher in aged neutrophils after stroke compared to monocytes (Fig. 2k, l). MMP-9 concentrations in the plasma were elevated both with age and after stroke, consistent with the protein levels detected in the ischemic hemisphere (SF5). These findings suggest a deleterious role for neutrophils in the ischemic brain of older mice, implicating bone marrow aging as a driving factor in stroke-induced neuroinflammation.

### Age positively correlates with neutrophil counts, MMP-9 expression, and hemorrhagic bleeding in postmortem brain tissue of acute ischemic stroke subjects

**Fig. 3:**
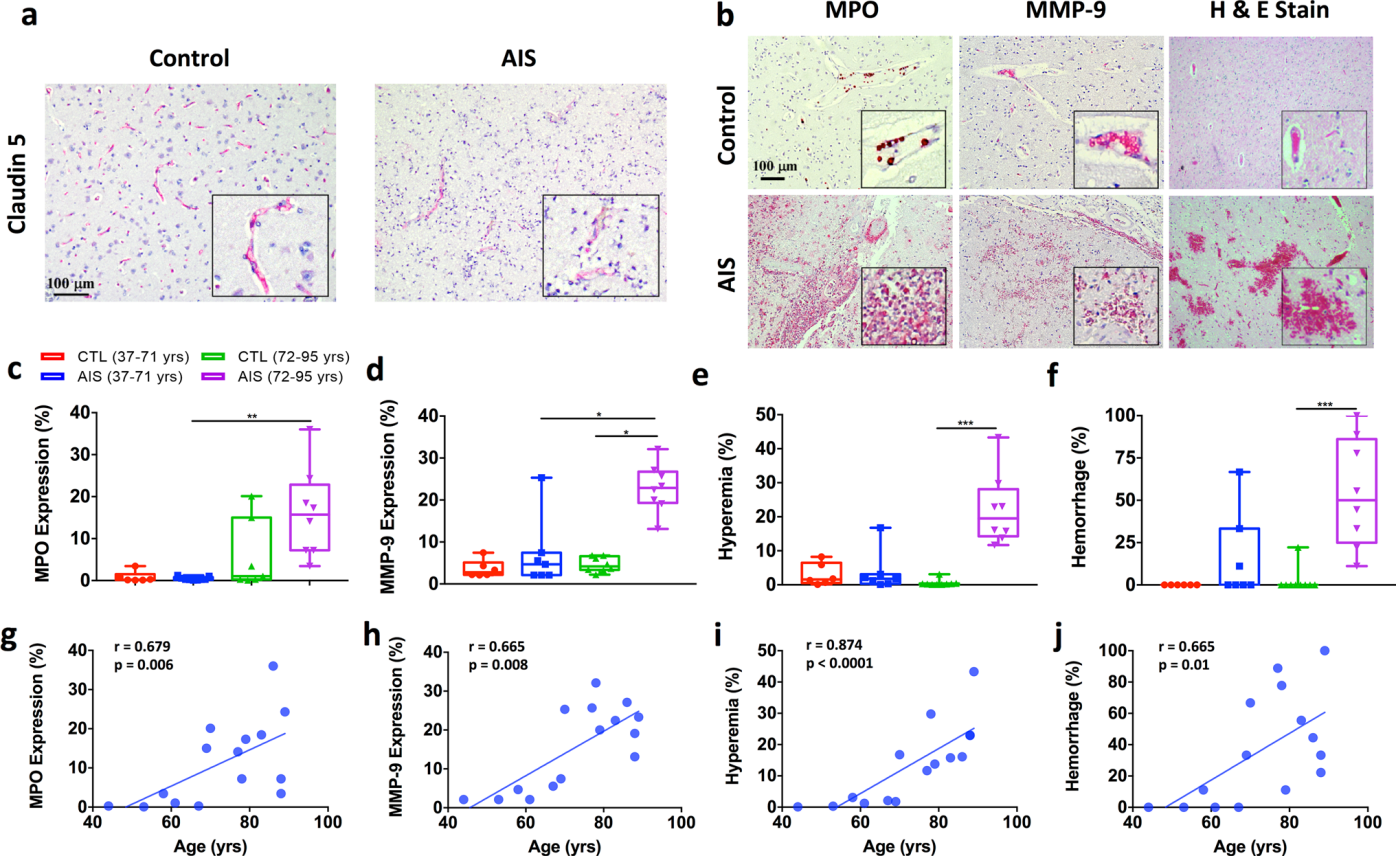
Higher neutrophil counts, MMP-9 expression, and hemorrhagic bleeding found in postmortem brain tissue of older acute ischemic stroke subjects. Representative immunohistochemistry microphotographs depicting claudin-5, myeloperoxidase (MPO), matrix metalloproteinase-9 (MMP-9), and hematoxylin and eosin (H&E) staining of postmortem human brain tissue from age-matched (> 60 years) control and acute ischemic stroke subjects (**a**, **b**, respectively; 10X, scale bar = 100 μm). The percentage area of MPO- and MMP-9-positive neutrophils was quantified (**c**, **d**, respectively; *N* = 6–9 control and 6–9 strokes/group). The percentage area in which hyperemia (**e**) and hemorrhage (**f**) was found significantly increased in older stroke subjects compared to age-matched controls relative to younger stroke subjects as determined by Kruskal–Wallis test with Dunn’s correction for multiple comparisons. Age correlations between MPO (**k**), MMP-9 (**l**), hyperemia (**m**), and hemorrhage (**n**) in stroke subjects (*N* = 15) were determined by Spearman correlation analysis. Error bars show mean SEM. *AIS* acute ischemic stroke, *H&E* hematoxylin and eosin and *SEM* standard error of mean. Dots represent individual subjects. *p < 0.05; **p < 0.01; ***p < 0.001

Table 1 summarizes the clinical characteristics of the 30 human postmortem cases with controls and stroke. The mean age was 71 years. There was no significant difference in the mean age between control (37–95 years, mean = 69.4 years) and stroke (44–89 years, mean = 72.7 years) groups. Postmortem brain tissue was acquired from mixed sex, age-matched control and ischemic stroke (53–89 years, mean = 74.0 years) individuals with acute/subacute stroke (7 days) and evaluated by immunohistochemistry. The relationship between neutrophil infiltration and vascular integrity after stroke was assessed in clinical stroke subjects. There was no correlation between infarct size and age (Table 2). Myeloperoxidase (MPO) staining identified hypochlorous acid-producing neutrophils in the brain, found both within claudin 5-stained vasculature and in the parenchyma, were significantly increased after stroke in old subjects (> 71 years old) compared to young (≤ 71 years old) stroke subjects (Fig. 3a–c). Expression of matrix metalloproteinase-9 (MMP-9), an enzyme involved in the degradation of the extracellular matrix, was significantly higher in old stroke subjects (> 71 years old) compared to both age-matched controls and young (≤ 71 years old) stroke subjects (Fig. 3b, d). High magnification images of hematoxylin and eosin (H&E)-stained and laminin alpha 5-stained sections demonstrated that many but not all neutrophils extravasated from vessels into the parenchyma (SF6). Hyperemia and hemorrhage were significantly increased in old stroke subjects compared to controls, whereas no statistical change was found in young subjects as determined by Kruskal–Wallis test (Fig. 3e, f, respectively). MMP-9 expression was associated with higher rates of MPO, hemorrhage, and hyperemia (Table 2). Age positively correlated with MPO and MMP-9 expression, hyperemia, and hemorrhage (Fig. 3g–j and Table 2). Together these findings support our experimental data, demonstrating robust neutrophil infiltration in the ischemic brain of older individuals that is associated with higher rates of hemorrhagic transformation.

**Table 1:** Demographic and characteristics of human postmortem cases

Characteristic	Control *N* = 15	Stroke *N* = 15	*P*
Age (year)	69.4 ± 15.3	72.7 ± 13.9	0.55^a^
Male, *n* (%)	8 (53.3)	7 (46.7)	0.72^b^
CAA stage	0(1)	1(3)	
None, *n*	11	5	
Mild, *n*	4	3	
Moderate, *n*	0	2	
Severe, *n*	0	4	
Hyperemia (%)	1.5 ± 2.4	13.5 ± 12.6	0.001^a^
Hemorrhage (%)	1.5 ± 5.7	36.3 ± 34.5	0.001^a^
MPO level (%)	0.6 ± 0.9	11.2 ± 10.7	0.001^a^
MMP-9 level (%)	4.2 ± 1.9	15.5 ± 10.6	0.001^a^
Infarct size (cm)	0	5.30 ± 2.8	
Treated with tPA, *n*	0	0	

*MPO* myeloperoxidase, *MMP-9* matrix metallopeptidase 9, *CAA* cerebral amyloid angiopathy, *tPA* tissue plasminogen activator

^a^Independent *t* test, mean ± standard deviation were reported

^b^ Mann–Whitney *U* test, median (interquartile range) was reported

**Table 2:** Correlation between different variables in the human postmortem cases of acute and subacute infarct ages

Parameters	Age	Hyperemia	Hemorrhage	MPO	MMP-9	Infarct size	CAA stage
Age	1.000						
Hyperemia	**0.874** ^**^	1.000					
Hemorrhage	**0.665** ^**^	**0.752** ^**^	1.000				
MPO	**0.697** ^**^	**0.629** ^*^	**0.788** ^**^	1.000			
MMP-9	**0.665** ^**^	**0.760** ^**^	**0.889** ^**^	**0.787** ^**^	1.000		
Infarct size	0.510	0.277	0.462	**0.692** ^**^	0.317	1.000	
CAA stage	− 0.024	0.169	0.289	0.110	0.426	− 0.344	1.000

Bold indicates statistically signficant correlations

*N* = 15. Spearman’s correlation. *p < 0.05, **p < 0.01

*MPO* myeloperoxidase, *MMP-9* matrix metallopeptidase 9, *CAA* cerebral amyloid angiopathy

### Differential effects of bone marrow aging on host brain production of growth factors, anti-inflammatory cytokines, and microglial function are seen in heterochronic chimeras

**Fig. 4:**
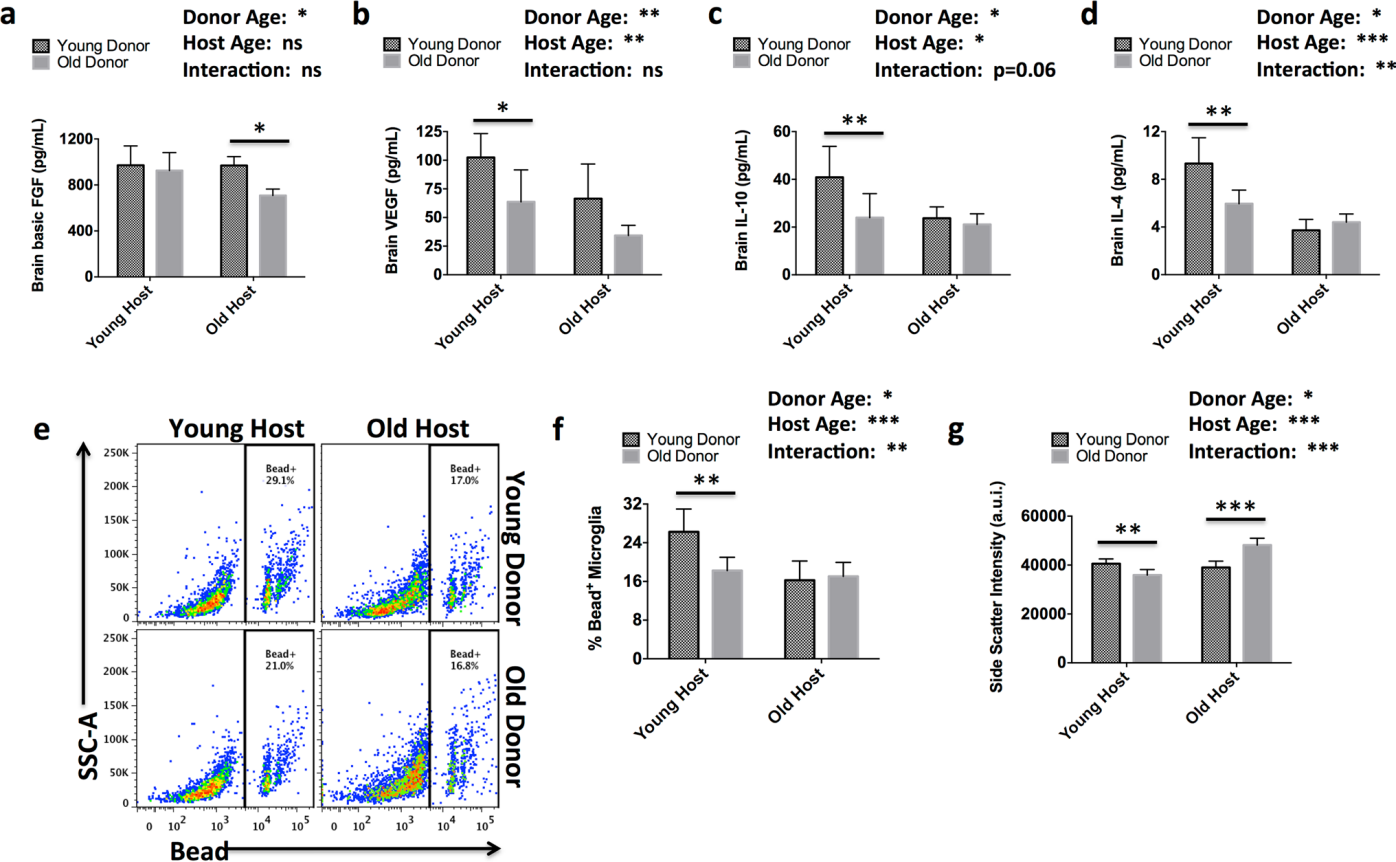
Age-dependent bone marrow regulation of brain immune factors is associated with altered microglia phagocytic activity. Brain protein concentrations of basic FGF, VEGF, IL-10, and IL-4 are shown for both heterochronic (Old → Young; Young→Old) and isochronic (Young → Young; Old → Old) bone marrow chimeric groups (**a**–**d**, respectively; *N* = 5–8/group). Microglia activity was measured using flow cytometry. Representative dot plots depict the percentage of microglia that have engulfed yellow-green fluorescent beads in an ex vivo phagocytosis assay (**e**; dotted = isochronic, solid = heterochronic, blue = young host, and red = old host). The percentage of bead-positive microglia was quantified (**f**; *N* = 7/group from a single experiment). Increased cellular granularity (side scatter) associated with the senescent phenotype was confirmed in older microglia as shown in isochronic groups, but was partially reversed in microglia from Young → Old chimeras (**g**). Error bars show mean SD. *a.u.i.* arbitrary units of intensity, *basic FGF* fibroblast growth factor, *VEGF* vascular endothelial growth factor, *IL-10* interleukin-10, *IL-4* interleukin-4, *SD* standard deviation. In **a**–**g**, statistical values were determined by two-way ANOVA with a follow-up Tukey multiple comparison test. Group effects are shown. *p < 0.05; **p < 0.01; ***p < 0.001

To investigate the possibility that either young monocyte promotes recovery or aged neutrophils hamper recovery following stroke, we generated heterochronic GFP bone marrow chimeric mice using young and old wildtype hosts. To determine whether the effects of aging on the bone marrow compartment contribute to normal CNS homeostasis we first measured the production of several growth factors and anti-inflammatory cytokines known to be associated with neuroprotection and regeneration. Aging peripheral immune cells differentially regulated brain production of these molecules (Fig. 4a–d). Aged heterochronic mice did not exhibit stereotypical age-related reductions in bFGF and VEGF, whereas young heterochronic mice displayed age-related reductions in IL-4 and IL-10 levels. Given the important role of debris clearance in CNS aging and disease, and that these molecules are tightly linked to microglia function, we next assessed phagocytic activity by the brain’s resident macrophage. Interestingly, old bone marrow-derived immune systems, independent of host age, significantly reduced the phagocytic potential of host microglia (Fig. 4e, f). Microglia from aged heterochronic mice had significantly lower dark granular content, which has been linked to the senescent phenotype (Fig. 4g). These results suggest that despite being confined by the blood–brain barrier, CNS homeostasis is highly influenced and sensitive to the effects of aging via systemic immune cells originating from the bone marrow.

### Bone marrow rejuvenation partially reverses age-related deficits in motor coordination, whereas old bone marrow promotes anxiety and depressive-like behavior in chimeric mice

**Fig. 5:**
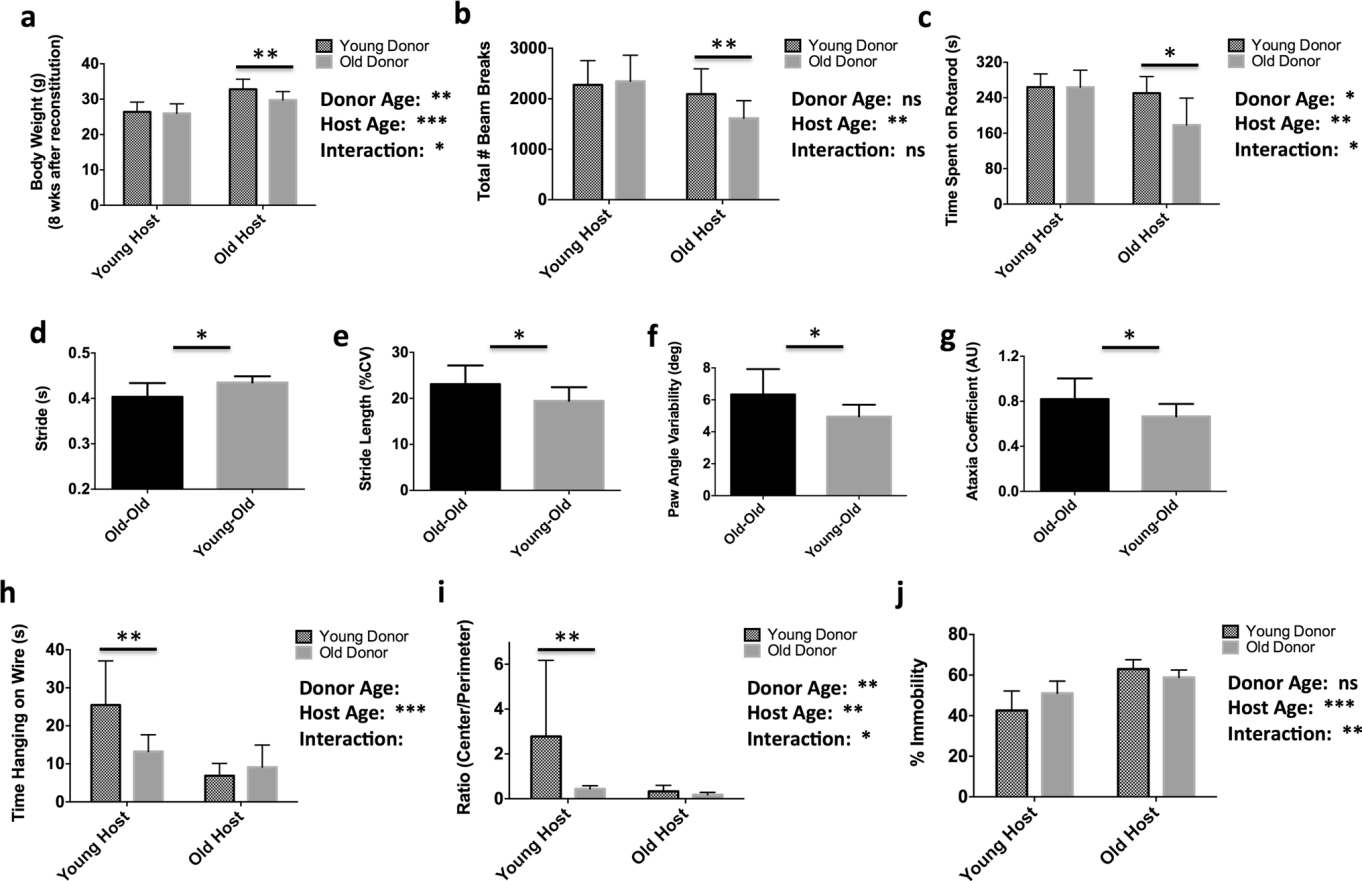
Heterochronic bone marrow transplantation partially reverses age-related frailty and modifies the neurobehavioral phenotype. Body weights were measured at 8 weeks following bone marrow reconstitution (**a**; *N* = 18–19/group). The total number of broken beams in an open field apparatus was measured over the course of 20 min (**b**). The latency to fall from an accelerating rotarod device is shown for each group (**c**). Digigait analysis was performed to assess changes in forelimb gait dynamics between Old → Old and Young → Old chimeras. Changes in stride time (**d**), stride length variability (**e**), paw angle variability (**f**), and ataxia (**g**) were evaluated (*N* = 9 and 10). Forelimb grip strength was measured by wire hang test and the latency to fall was quantified (**h**). The relative amount of time spent in the center quadrant of the open field apparatus versus in the surrounding perimeter during the 20-min session is shown (**i**). For all behavior experiments, *N* = 8–10/group. The percentage of time spent immobile during 5 min of a tail suspension test was quantified (**j**). Error bars show mean SD. *SD* standard deviation. In **a**–**c** and **h**–**n**, statistical values were determined by two-way ANOVA with a follow-up Tukey multiple comparison test. Group effects are shown. In **d**–**g**, statistical analysis was performed by unpaired, two-tailed Student’s *t* test. *p < 0.05; **p < 0.01; ***p < 0.001

Because changes in brain inflammatory activity are generally correlated with behavioral alterations [97], we then examined the effect of aging immune systems on the behavioral phenotype of recipient mice. Although there were no differences during the first 5 weeks of reconstitution (data not shown), body weights began to diverge at 8 weeks. Aged heterochronic mice weighed significantly more than isochronic controls, consistent with attenuation in the age-related frailty phenotype (Fig. 5a). Bone marrow rejuvenation rescued age-related deficits in general locomotor activity and motor coordination as evidenced by total beam breaks in the open field (Fig. 5b) and time spent on an accelerating rotarod (Fig. 5c). Moreover, aged heterochronic mice exhibited significantly improved gait dynamics compared to isochronic controls (Fig. 5d–g). Conversely, young heterochronic mice showed decreased forelimb grip strength (Fig. 5h), spent significantly longer times near the perimeter of the open field (Fig. 5i), and displayed less mobility in the tail suspension test (Fig. 5j). Taken together, bone marrow rejuvenation attenuated several features of age-related frailty and disability, whereas the effects of old bone marrow-derived immune systems have the potential to induce an age-associated anxiety-depressive-like phenotype in young hosts.

### Old bone marrow augments the infiltration of neutrophils into the CNS independently of infarct size

**Fig. 6:**
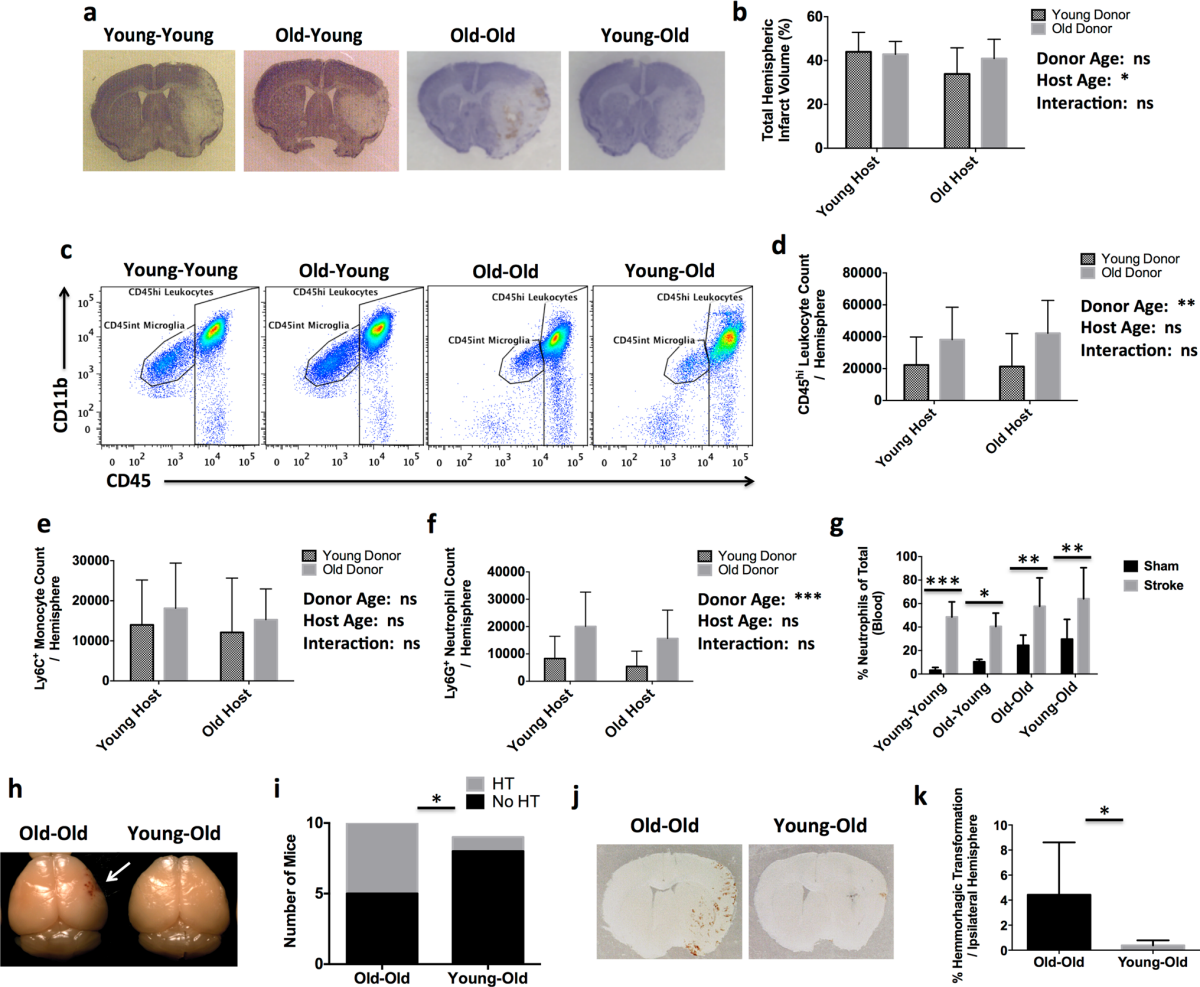
Heterochronic bone marrow transplantation does not regulate infarct size after stroke but does exacerbate neutrophil infiltration and hemorrhagic transformation in chimeric mice. Representative images of cresyl violet-stained coronal brain sections for heterochronic and isochronic bone marrow chimeric mice at 72 h after middle cerebral artery occlusion (**a**). Infarct volumes were measured and the total ipsilateral hemispheric area relative to the contralateral side is shown (**b**; *N* = 9–13/group). Representative dot plots depict the extent of CD45^hi^CD11b^+^ myeloid cell infiltration in the ischemic brain of each group (**c**). The absolute number of total infiltrating CD45^hi^ leukocytes was quantified (**d**) and the number of monocytes (CD45^hi^CD11b^+^Ly6C^+^Ly6G^−^) and neutrophils (CD45^hi^CD11b^+^Ly6C^+^Ly6G^+^) were compared (**e** and **f**, respectively). The frequency of neutrophils in the blood was quantified in sham and stroke chimeras (**g**). For **e**, **f**, and **g**, N = 8–16/group. Gross examination revealed higher rates of hemorrhagic transformation in Old → Old chimera brains at 72 h after stroke compared to Young → Old chimeras (**h**, **i**; *N* = 9 and 10). Representative histological sections show petechial bleeding in the post-ischemic brain (**j**). The percentage of area covered by hemorrhagic transformation in the ipsilateral hemisphere was measured quantitatively (**k**; *N* = 6 and 6). Error bars show mean SD. *ST* stroke; *SD* standard deviation. In **b** and **d**–**f**, statistical values were determined by two-way ANOVA with a follow-up Tukey multiple comparison test. Group effects are shown. In **i**, data were analyzed by two-tailed, Chi-square test. In **k**, statistical analysis was performed by unpaired, two-tailed Student’s *t* test. *p < 0.05; **p < 0.01; ***p < 0.001

Our results show that cellular components of the young immune system have the potential to reverse age-related neurobehavioral deficits in old chimeric mice, whereas replacement of an old immune system increases vulnerability of young chimeric mice to impaired CNS homeostasis and negative affective states. We hypothesized that young bone marrow responses promote a beneficial neuroinflammatory milieu in the ischemic brain by biasing recruitment of injury-resolving monocytes. No effect of donor age was seen on infarct size. However, group analysis revealed a significant effect of host age on infarct volume, with old hosts having smaller infarct volumes and less body weight loss and splenic atrophy (Y → Y, 2.316 ± 0.11; O → Y, 2.409 ± 0.12; O → O, 1.866 ± 0.15; and Y → O, 1.982 ± 0.13 mg/g body weight) than young hosts after stroke, independent of bone marrow age (Fig. 6a, b). Although no changes in tissue injury were detected, a significant increase in the number of total brain-infiltrating CD45^hi^ leukocytes derived from old bone marrow was found (Fig. 6c, d). Monocyte counts were similar between all groups (Fig. 6e), but there was a significant increase in the number of old neutrophils in the ischemic brain of young heterochronic and old isochronic mice (Fig. 6f). Blood neutrophilia was evident in all chimera groups after stroke and a significant effect of host age was seen (Fig. 6g). To see if these features of neutrophil activation were associated with age-related increases in the rate of hemorrhagic transformation after stroke (after normalizing for infarct volume, see Fig. 1c) we examined the total area of hemoglobin oxidation present within the ipsilateral hemisphere. Higher rates of petechial bleeding in isochronic controls compared to Young → Old mice were seen (Fig. 6h, i). Aged heterochronic mice exhibited significantly less hemorrhagic transformation than isochronic controls (Fig. 6j, k). These results suggest that the mechanisms mediating histological infarct size in the ischemic brain are largely dependent on the response of the host CNS environment, as they were unaltered by bone marrow replacement, whereas the degree and cellular composition of the infiltrating leukocytes are dependent on the age of the bone marrow compartment. These findings shifted our focus from young monocytes to the detrimental interaction between age and ischemia on neutrophil responses as the potential driver of increased age-related stroke severity.

### Bone marrow rejuvenation reduces the severity of acute behavioral deficits following stroke

**Fig. 7:**
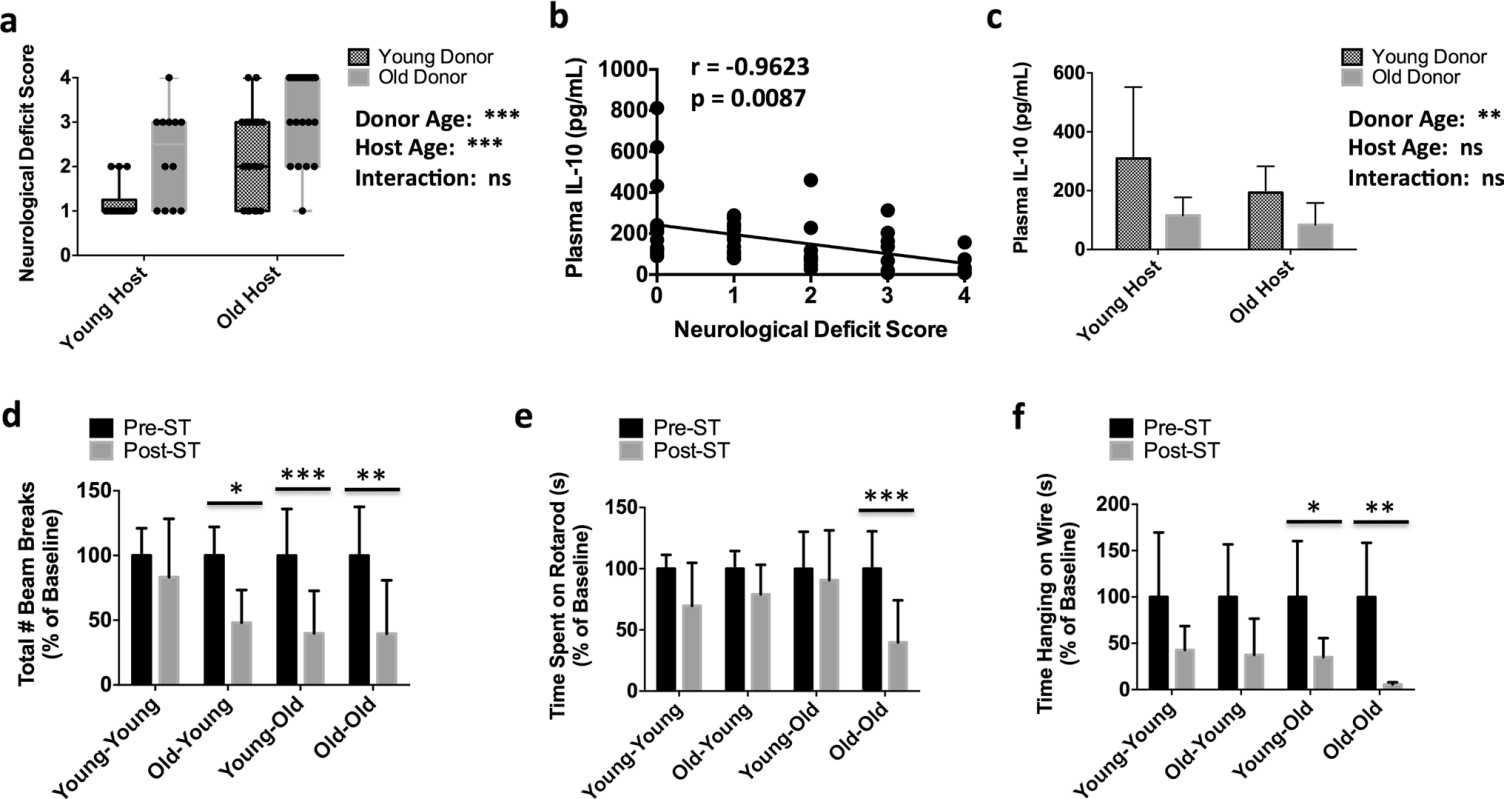
Bone marrow rejuvenation improves acute behavioral outcomes after stroke in old chimeric mice. The neurological deficit scores were assessed for each group at 72 h (**a**; *N* = 12–22/group). A correlation analysis was performed between neurological deficit scores and plasma IL-10 protein concentrations taken at 72 h (**b**; Pearson Correlation, *p* < 0.01; *N* = 49). Plasma IL-10 concentrations were measured by ELISA at 72 h after stroke and quantified using two-way ANOVA (**c**; *N* = 8–10/group). Behavioral outcome was characterized at 72 h after stroke using open-field testing (**d**), rotarod testing (**e**), and wire hang testing (**f**) to determine spontaneous locomotor activity, balance and motor coordination, and forelimb grip strength, respectively (*N* = 9–11/group). Error bars show mean SD. *ST* stroke; *SD* standard deviation. Dots represent individual mice. In **b**, correlation analysis was performed using Spearman’s correlation coefficient. In **c**, statistical values were determined by two-way ANOVA with post hoc Tukey test for multiple comparisons. Group effects are shown. In **d**–**f**, data were analyzed using two-way ANOVA with Sidak’s multiple comparison test. *p < 0.05; **p < 0.01; ***p < 0.001

Next we determined whether bone marrow rejuvenation, and its resultant attenuation in neutrophil recruitment, was associated with any behavioral changes following stroke. No differences in mortality rates were found between groups (data not shown). A significant effect of bone marrow aging was found for clinical score, with old heterochronic mice displaying significantly lower neurological deficit scores at 72 h after reperfusion than isochronic controls (Fig. 7a). We then found that plasma IL-10 levels negatively correlated with neurological outcome at 72 h, which was further highlighted by the differential response in both heterochronic groups (Fig. 7b, c). Behavioral evaluation at 72 h demonstrated significant deficits in Old → Young mice compared to isochronic controls (Fig. 7d). Bone marrow rejuvenation improved rotarod performance in Young → Old mice compared to isochronic controls (Fig. 7e). The percent change from baseline in Young → Old mice was similar to Young → Young mice but significantly less than that seen in Old → Old mice after stroke (35.0 vs. 5.57 s, *p *< 0.05, Fig. 7f). Together these results suggest that the effects of aging on the bone marrow response to brain injury result in neutrophil-biased recruitment, leading to worse behavioral outcomes following stroke.

## Discussion

This study demonstrates that features of age-related frailty at baseline, as well as motor recovery processes following stroke, are highly dependent on the age and status of the immune system. The processes that underlie aging and inflammation are inextricably linked and form the conceptual basis of ‘inflamm-aging’, the phenomenon that describes the subtle increase in inflammatory stress with advanced age. In this study, we show that the immune response to stroke differs and is dependent on the age of the animal examined. Stroke leads to the predominate recruitment of monocytes in young mice and of neutrophils in aged mice. In human postmortem brain tissue, age positively correlated with neutrophil infiltration, MMP-9 expression, and hemorrhage after ischemic stroke. While these findings may reflect other factors present in older patients such as cerebral amyloid angiopathy, we found no correlation with the severity of pathological cerebral amyloid and hemorrhage, hyperemia or neutrophil infiltration (Table 2). Experimentally, the neutrophilic response was associated with poor behavioral outcomes and hemorrhagic transformation in aged mice. Relative to brain-infiltrating monocyte populations, stroke-responsive neutrophils produced more MMP-9 and ROS, and had a reduced capacity for phagocytosis which was further exacerbated with age. In this work, we have also highlighted the utility of flow cytometry for the identification of subpopulations of resident microglia, demonstrating marked heterogeneity in the microglial response to brain injury that implies that subsets of senescent or dystrophic microglia may be key drivers of injury [82]. Dystrophic Side Scatter^hi^ populations of aged microglia display higher ROS production and an inability to produce pro-inflammatory cytokines after stroke.

Manipulation of the immune system attenuates the negative effects of aging in a heterochronic bone marrow chimera model. Surgical parabiosis studies in which young and aged mice share common circulation have highlighted the potential of youthful plasma factors to partially reverse age-related deficits in neurogenesis, remyelination, and muscle regeneration [41, 70, 79, 92]. Conversely, several blood-borne factors identified as both pro-aging and pro-inflammatory are elevated with age and contribute to cognitive impairment [59, 80, 91]. While these pioneering studies were the first to demonstrate the profound effect of the systemic environment on brain function, the cellular origin of these blood-borne factors is unknown. In this work we show that CNS rejuvenating effects can be recapitulated by heterochronic bone marrow transplantation, suggesting a novel immune-based strategy for reversing age-related deficits. Bone marrow rejuvenation partially ameliorated several features of age-related frailty in uninjured mice and promoted an immune environment that favors protection after stroke and a reduction in hemorrhagic transformation. These immune-driven effects may have broad implications for both brain and systemic aging, as a link between stress-induced bone marrow production of myeloid cells and cardiovascular disease risk has recently been established [32].

The use of heterochronic parabiosis has aided our understanding of the rejuvenation process [42]. However, given its self-renewing capacity, bone marrow manipulation may be a more practical method of reverse-engineering the effects of aging. Bone marrow rejuvenation increases angiogenic growth factor production and restores age-related deficits in angiogenesis and cardiac function after stress and injury [29, 46, 81], benefits that persisted up to a year. Heterochronic transplants also mitigate renal aging and increase bone mineral mass and fracture repair [3, 98, 101]. Together, these results imply a profound global improvement in systemic function following immune reconstitution. Interestingly, we found significant cognitive deficits in young mice that received aged bone marrow, suggesting that there is a detrimental factor produced by aged immune cells that is transmissible. Aged bone marrow also decreased growth factor expression, microglia phagocytosis and led to an anxiety/depressive phenotype in young mice. Recent findings have shown that repeated heterochronic bone marrow transplantation without additional conditioning (i.e., irradiation) can extend lifespan by 34% in syngeneic hosts; however, the effects on inflammation, health span and frailty were not addressed [40, 43].

Frailty is a common clinical syndrome in the elderly that carries an increased risk for poor health outcomes including falls, disability, and mortality [100]. According to Fried et al [26], frailty is defined by a set of criteria that includes: low energy and physical activity, impaired gait dynamics and grip strength, and unintentional weight loss. Our data emphasizes the importance of systemic immunity to overall health. Our studies imply that bone marrow manipulation by rejuvenation of the HSC pool provides chronic restoration of function and eliminates the need for repeated acute treatments of young blood.

Numerous experimental studies have shown that adoptive transfer of bone marrow stem cells shortly following ischemia has neuroprotective and anti-inflammatory benefits [4]. Although our study demonstrates the importance of prophylactic bone marrow transplantation in mediating behavioral and neurorepair processes, the potential for bone marrow rejuvenation as a post-reparative therapy requires further study and has considerable obstacles such as donor source and rejection. Although there have been major advances in transplantation therapies for CNS injuries such as stroke, major limitations (engraftment, differentiation, functional incorporation) and hurdles (long-term benefit) still exist. Perhaps our most interesting and novel finding is that youthful bone marrow-derived immune cells exerted a rejuvenating effect on the aged CNS despite the continued presence of aged blood vessels, neurons, and glia as evidenced by higher anti-inflammatory cytokine levels, attenuated microglial senescence phenotypes, and improved motor function and gait dynamics. Previous work using heterochronic chimeras has demonstrated that bone marrow aging leads to the pathological accumulation of macrophages and increased fibrosis in muscle tissue [96], which could partly explain the neurological benefits seen in the present study. However, the poorer neurological performance in young mice (hosts) that are reconstituted with aged marrow suggests it is not entirely the muscles that are the issue, as the host is still young, except for the aged immune system. Bone marrow transplantation may remove and replace senescent bone marrow HSCs with younger, healthier ones. The requirement for bone marrow transplantation itself could be fully avoided if drugs can effectively and safely target and eliminate aged cells, allowing young, healthy cells to take over this niche. Ongoing studies are evaluating the specific cell types responsible for the rejuvenating effects of bone marrow replacement in an attempt to further refine therapeutics.

Microglia are important regulators of CNS homeostasis in both health and disease. Our study found that hemispheric microglia numbers were significantly lower in old mice. This is in line with previous studies showing 40% reductions in cortical microglia in histological preparations and 25% reductions using flow cytometry [75, 103]. It is reasonable to consider that like many other cells in the aged brain (i.e., oligodendrocytes, neurons, neural progenitors, etc.), there are similar reductions in microglia number, especially as recent studies suggest that these cells may have a finite lifespan. As noted by others [83], aged microglia were significantly more granular and complex compared to their younger counterparts, suggesting a conversion to this appearance and morphology occurs over time. Following stroke, aged microglia were found to be strong producers of ROS, particularly the highly granular ‘dystrophic’ populations. Although global ROS levels were not measured in this study, it is likely that the young brain, with its larger infarct volume and greater cell infiltration, would stand to be comparably greater than old ischemic brain. Given that previous studies have shown that aged neurons are more vulnerable to oxidative stress [25, 99], the fact that microglia from old mice have higher ROS levels at baseline and at day 3 may help us understand the poor long-term recovery in aged mice compared to young, which resolves stroke-induced inflammation sooner. Indeed, aged microglia are ‘primed’ to have an exaggerated response following injury [56]. Moreover, the extent to which this microglial response is driven by dystrophic or senescent microglia populations versus the co-existing ramified populations is not known. The novel application of side scatter gating to identify ‘dystrophic’ microglia was exploited for this purpose, which does not require additional antibodies, may allow us to understand how these cells differ from their ramified counterparts and determine their role in neurodegenerative diseases and stroke recovery.

Our data also highlight the deleterious role of aging in the biased production of bone marrow-derived myeloid subsets after injury. Neutrophils rapidly migrate into injured tissues and directly attack microorganisms. In sterile inflammatory responses, such as ischemic stroke, the same functions (i.e., respiratory burst) that make them an effective defense against pathogens cause a significant amount of bystander damage. Studies have shown a detrimental role for neutrophils in human stroke patients [33, 38, 50], and neutrophil activation and hemorrhagic transformation via MMP production. Inhibition of MMP-9, the primary MMP implicated in stroke-induced neuroinflammation and the biphasic disruption of the blood–brain barrier, has shown mixed results in experimental studies depending on the timing of administration [102]. Early treatment (3–24 h) has demonstrated robust neuroprotection and preservation of the BBB, whereas delayed treatment (72 h–7 days) has shown either no effect or exacerbation of stroke pathology [10]. Thus, the temporal aspects of vascular remodeling are complex, but suggest that MMP-9 activity may actually be beneficial at later stages of injury. Although neutrophils are generally believed to be the first to enter the brain early after an ischemic event, the prolonged presence of these cells 3 days later in old mice, led to poorer outcomes. Interestingly, in the light of the recent report that neutrophils are restricted within luminal surfaces or perivascular spaces of cerebral vessels following ischemic injury, our study found evidence for neutrophil extravasation into parenchymal areas outside of the basement membrane [22]. Although CCL5/RANTES concentrations in the brain and plasma were higher in old mice after stroke, leukocyte infiltration was substantially lower, suggesting the possibility of age-related defects in migratory ability [18]. This finding is consistent with an earlier study that showed higher CCL5 protein concentrations in the brain of old mice at 48 h after MCAO compared to adult mice [77]. The authors noted that the inflammatory profile in the ischemic brain was profoundly altered with age [78], and several pro-inflammatory cytokines were elevated despite the smaller infarcts seen in older mice. Moreover, other studies have reported both very low levels and modest or no change in CCL5 expression in young mice at 6 h following injury [16, 77]. Age-related increases in CCL5 have been previously reported by others and suggest the possibility that neutrophil recruitment may be augmented or altered in old mice following stroke, and this response may not be seen in the young. Previous work has demonstrated a link between circulating CCL5 levels, neutrophil invasion, and the poorer stroke outcome in aged mice; however, this was studied only in the context of chronic peripheral infection [16, 19, 85]. Our study underscores the interaction between normal aging and ischemic injury on leukocyte responses. Aged neutrophils in the ischemic brain exhibit deficits in debris clearance, exacerbated oxidative stress levels, and augmented production of enzymes responsible for vascular remodeling and blood–brain barrier permeability. Aging exacerbates the frequency and degree of hemorrhagic bleeding subsequent to thrombolysis [21] and recent reports have found that neutrophil counts predict bleeding risk following TPA administration [50]. Our study also suggests a pro-restorative (or less deleterious) role for monocytes after stroke in the aged brain. Whereas the role of Ly6C^lo^ monocytes appears to be redundant [54], recent evidence supports the notion that Ly6C^hi^ monocytes exert an acute protective role following stroke by promoting M2 macrophage polarization and preventing hemorrhagic transformation [12, 28]. The protective responses of monocytes in young ischemic brains may be secondary to their high capacity to phagocytose and clear dying pro-inflammatory neutrophils and other debris. While CCL2 concentrations were significantly similarly augmented in the acute ischemic brain, the cellular source of this chemokine was not assessed. Previous studies support a strong role for endothelial cells and astrocytes as important mediators of monocyte recruitment [37, 84]. Our findings also highlight the importance of modeling stroke injury in aged mice as the finding of this biased recruitment of neutrophils in older mice represents an important translational advance for therapeutic discovery that would not have been seen if only young mice were evaluated.

Our finding that neutrophilia is exacerbated with age is consistent with normal age-related myeloid biasing of bone marrow cell production. While it is still unclear whether age is associated with greater severity of stroke-induced neutrophilia in humans, our data are consistent with recent reports that demonstrated that higher circulating neutrophil counts are independently associated with symptomatic intracerebral hemorrhages and worse outcomes at 3 months [50]. This is not surprising given that neutrophils are the strongest producers of reactive oxygen species and MMPs, two potent drivers of neuronal injury and blood–brain barrier disruption, respectively [2, 68]. Indeed, higher serum levels of MMP-9 at stroke onset have been shown to predict poor outcome in the acute period [1]. Interestingly, while neutrophil invasion of ischemic brain tissue has been previously shown in stroke patients [58, 67, 68], our histological evidence provides an even stronger relationship between aging and neutrophil counts, co-localized production of extracellular matrix-degrading enzymes (i.e., MMP-9), and increased rates of hemorrhagic bleeding in the brain as a larger cohort was examined. The finding that neutrophil extravasation is elevated in ischemic regions of older clinical subjects may simply reflect species-related differences in immunology, as neutrophils are far more abundant in human blood than in mice (10–25% mice vs. 60–70% human) [104]. However, the effects of aging on myeloid biasing and neutrophil function are highly conserved between mice and men, suggesting that the ‘biological’ age of the immune system (i.e., neutrophils) may be a better predictor of stroke outcomes rather than ‘chronological’ age. Taken together, these data further support the use of aged rodent models of ischemic stroke to better reflect the neutrophilic response to brain injury observed in patients.

Despite this innovative approach to evaluate the central and systemic roles of brain aging/injury, this study has several limitations. As we did not directly examine non-irradiated mice, interpretation of our results may be confounded by differences in the ability of young and old mice to recover from irradiation. This is unlikely as the rejuvenation (i.e., Young → Old) and age-acceleration (i.e., Old → Young) patterns pertaining to brain production of cytokines and growth factors [57, 76], microglia phagocytosis [65], locomotor activity and coordination [23, 24], emotional behavior [13, 27, 87], and other features of frailty [49] are highly consistent with the literature in aging research. Despite these findings, it is worth noting that significant interactions between age and irradiation in microglia activation have been demonstrated by others [36, 45, 73]. Additionally, chronic time points are required to understand how this acute neutrophil response can affect delayed lymphocyte recruitment, recovery, cognitive function, and glial scar formation [20]. Aged mice had significantly fewer infiltrating monocytes after stroke compared to young mice. Given the wound-healing role for monocytes, this attenuation in the monocytic response, rather than an enhanced neutrophilic response, may provide a better explanation for these worsened outcomes. However, given the difficulty in distinguishing monocytes/macrophages from resident microglia in the brain using standard histological procedures, it is not clear to what extent monocyte infiltration is affected with age in humans. Although the timing of these responses may dynamically change with age, we have demonstrated that leukocyte infiltration in aged animals can be altered with substantial benefit to behavioral outcome. Finally, outcomes were assessed using only one experimental model of stroke. Future investigations should investigate other models including a permanent MCAO or gradual reperfusion model which could have greater translational relevance [34]. However, the heterochronic bone marrow model system in this study was not used as a precursor to a therapeutic strategy, but was utilized to improve our understanding of how systemic immune system aging contributes to stroke outcomes. In addition, our experimental paradigm in which bone marrow rejuvenation precedes stroke-induced injury presents a translational barrier to treatment timing, as stroke is an unpredictable occurrence in humans. However, the beneficial effects seen in uninjured animals suggest that similar therapeutic applications could be both prophylactic and of wider use to the much larger disabled, frail elderly population for which there are no treatment options.

In conclusion, we have demonstrated that the immunological response to stroke differs in young and aged mice, with a profoundly skewed neutrophil response in the aged. While host age (i.e., the native brain environment) was found to be a major determinant of infarct size, age-related changes in the peripheral immune response to stroke directly exacerbated neuroinflammation and led to worse behavioral deficits and poorer recovery that were independent of infarct size. These findings mirrored our human data, which demonstrated that age positively correlates with neutrophil infiltration and hemorrhage, but not infarct size. While these results are surprising and provocative, numerous studies have now demonstrated that brain inflammation and behavior can be altered independently of histological damage/infarct size [30, 53, 74, 89]. Bone marrow rejuvenation benefited uninjured aged mice, both in terms of enhanced mobility and strength. This suggests that experimental strategies to replace senescent bone marrow may bestow health benefits to many elderly individuals, even in the absence of an acute injury. This work also demonstrates that there is an age-related bias in leukocyte recruitment to the ischemic brain. This is associated with significantly exacerbated behavioral deficits and increased hemorrhagic transformation following ischemic stroke. This was partially mitigated by bone marrow rejuvenation despite the continued presence of aged vasculature, neurons, and glia in the host. Taken together, we have demonstrated that bone marrow aging is responsible for many of the stereotypical age-related features of CNS senescence in a young host, whereas young bone marrow can promote healthy motor function and more improves the response to ischemic brain injury. Given its homeostatic role in the performance of many of the body’s tissue maintenance and repair functions, the potential for bone marrow rejuvenation holds great promise for the development of the next-generation of age-related therapies.

## Electronic supplementary material

Below is the link to the electronic supplementary material.

***Supplemental Figure 1. Effect of aging on TNF production in infiltrating Ly6C***^***+***^ ***monocytes after stroke.*** *A representative dot plot shows the percentage of TNF-positive Ly6C+ monocytes in the ischemic brain of young and old mice at 72 hrs (****a****). No significant difference was found between groups using Student’s t-test (****b****; N=6/group)). Error bars show mean SD. Abbreviation: ns not significant, SD standard deviation, SSC side scatter, ST stroke, TNF tumor necrosis factor.* (TIFF 1014 kb)

**Supplemental Figure 2. Aged microglia exhibit higher cellular granularity which is associated with exaggerated functional responses after stroke.** Representative dot plots illustrate the relative level of cellular granularity and complexity (side scatter axis) in aged microglia compared to young (**a**). A distinct population of side scatter (SSC)^hi^ microglia in aged mice exhibit attenuated production of TNF at 72 hrs after stroke compared to young and aged SSC^low^ populations (**b**). Quantification of the mean fluorescence intensity of DHR123^+^ microglia demonstrate comparably higher ROS production in aged SSC^hi^ microglia relative to SSC^low^ populations (**c**). Error bars show mean SD. Abbreviation: HI high, SSC side scatter, TNF tumor necrosis factor, DHR123 dihydrorhodamine 123, SD standard deviation. In **b** and **c**, statistical values were determined by two-way ANOVA with a follow-up Tukey multiple comparison test. *p<0.05; ***p<0.001 (TIFF 482 kb)

**Supplemental Figure 3. Effect of age on CCL2 and CCL5 concentrations after ischemic stroke.** Plasma and brain concentrations of the chemokine CCL2 were elevated with age and after stroke in old mice compared to young mice (**a** and **b**, respectively; N=5-7/group). A significant effect of age (F(1, 30)=9.447, P=0.0045) and stroke (F(2, 32)=10.09, P=0.0004) was seen on CCL2 levels in the plasma and brain, respectively. CCL2 protein was significantly higher in the brain at 72 hrs after stroke relative to sham compared to young as determined by two-way ANOVA with post-hoc Tukey test for multiple comparisons. Plasma and brain concentrations of the chemokine RANTES/CCL5 were elevated with age and after stroke in old mice compared to young mice (**a** and **b**, respectively; N=5-7/group). CCL5 protein was significantly higher in old plasma and brain at 72 hrs after stroke compared to young as denoted by #. Error bars show mean SD. Abbreviation: CCL2 chemokine (C-C motif) ligand 2, SD standard deviation. **p<0.01 (TIFF 434 kb)

***Supplemental Figure 4. Age-related changes in brain-infiltrating leukocyte composition after stroke.*** *Pie charts illustrate significant infiltration of cells of myeloid origin in both young (****a****) and aged (****b****) ischemic brains at 72 hrs in a cohort of injured animals. Compositional analysis of this bulk myeloid population reveal a substantial age-related bias in specific subsets found in young (****c****) and old (****d****) mice, including Ly6C*^*+*^ *monocytes and Ly6G*^*+*^ *neutrophils.* (TIFF 331 kb)

***Supplemental Figure 5. Plasma and brain MMP-9 concentrations in aging mice after ischemic stroke.*** *Plasma concentrations of MMP-9 were measured in young and old mice at 72 hrs after stroke (****a****). Concentrations were analyzed by two-way ANOVA with post-hoc Tukey test for multiple comparisons (N=4/group). Brain concentrations of MMP-9 are shown for the ischemic brain and analyzed by unpaired, two-tailed Student’s t-test (****b***; N=4/group). Error bars show mean SD. Abbreviation: mL milliliter, MMP-9 matrix metalloproteinase-9, ng nanogram, SD standard deviation, ST stroke. *p<0.05; **p<0.01; ***p<0.001 (TIFF 260 kb)

***Supplemental Figure 6. Histological assessment of neutrophil extravasation into the parenchyma after stroke in humans.*** *Observational evidence for the transmigration of neutrophils into brain parenchyma was determined by H & E staining and immunohistochemistry of post-mortem human brain tissue from acute ischemic stroke subjects. Higher magnification of H & E staining shows congested capillaries (visible as bright red lines in the image) with a dense cluster of neutrophils in between the vessels (****a****). There are also a few residual dead-red ischemic neurons in the picture. Immunostaining for laminin alpha 5, a component of the endothelial basement membrane, shows that neutrophils are found in parenchymal areas outside of the basement membrane (****b****; 20X, scale bar = 250 μm). Red arrows indicate MPO-positive neutrophils and black arrows indicate endothelial basement membrane. Abbreviation: H & E hematoxylin and eosin.* (TIFF 5141 kb)
