# Supplementary figures and images for: Aging alters the immunological response to ischemic stroke
Source: Acta Neuropathol. 2018 May 11;136(1):89–110. doi: 10.1007/s00401-018-1859-2 (PMC6015099; doi:10.1007/s00401-018-1859-2)

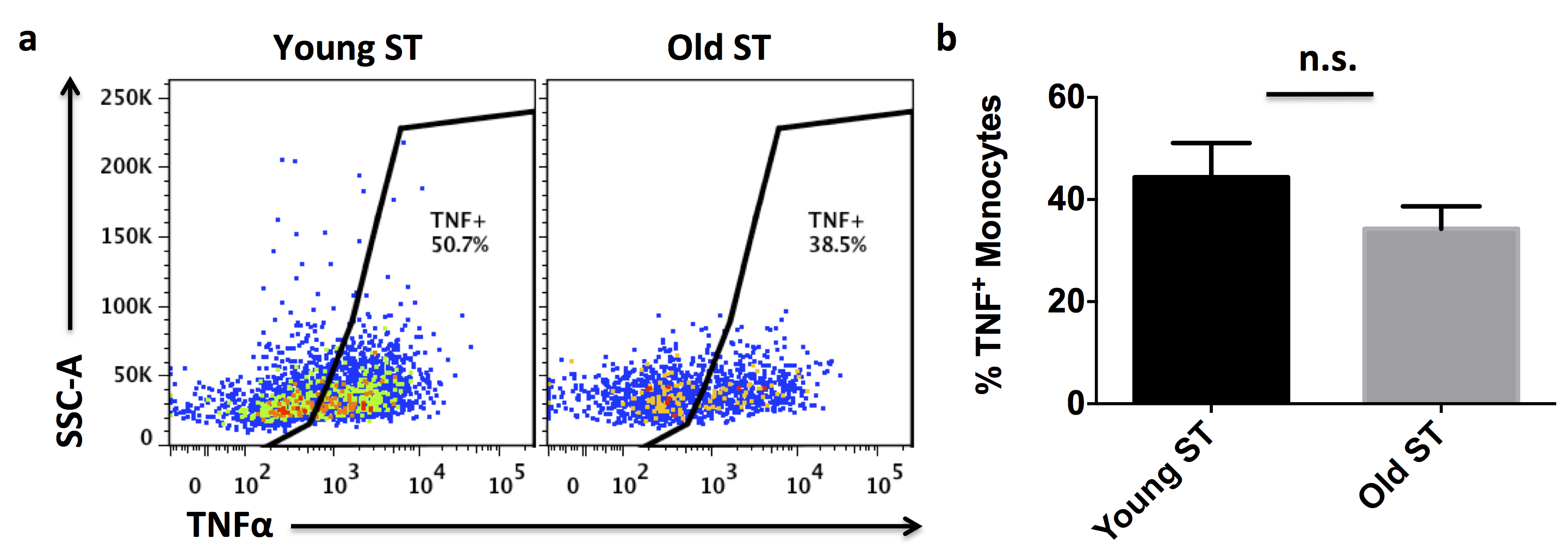

Supplement: Supplementary file 1 — Supplemental Figure 1. Effect of aging on TNF production in infiltrating Ly6C+ monocytes after stroke. A representative dot plot shows the percentage of TNF-positive Ly6C+ monocytes in the ischemic brain of young and old mice at 72 hrs (a). No significant difference was found between groups using Student’s t-test (b; N=6/group)). Error bars show mean SD. Abbreviation: ns not significant, SD standard deviation, SSC side scatter, ST stroke, TNF tumor necrosis factor. (TIFF 1014 kb) [file 401_2018_1859_MOESM1_ESM.tiff]

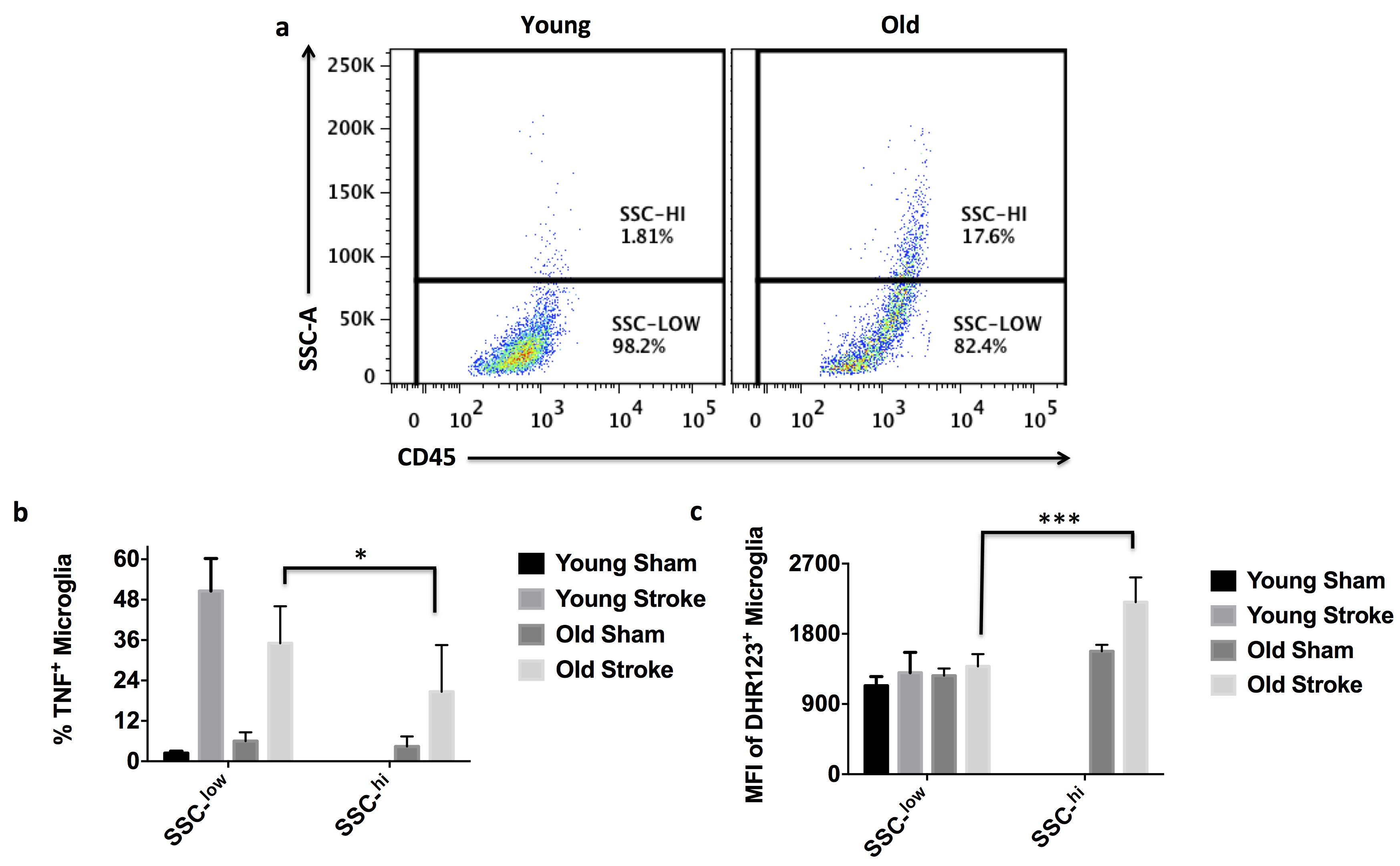

Supplement: Supplementary file 2 — Supplemental Figure 2. Aged microglia exhibit higher cellular granularity which is associated with exaggerated functional responses after stroke. Representative dot plots illustrate the relative level of cellular granularity and complexity (side scatter axis) in aged microglia compared to young (a). A distinct population of side scatter (SSC)hi microglia in aged mice exhibit attenuated production of TNF at 72 hrs after stroke compared to young and aged SSClow populations (b). Quantification of the mean fluorescence intensity of DHR123+ microglia demonstrate comparably higher ROS production in aged SSChi microglia relative to SSClow populations (c). Error bars show mean SD. Abbreviation: HI high, SSC side scatter, TNF tumor necrosis factor, DHR123 dihydrorhodamine 123, SD standard deviation. In b and c, statistical values were determined by two-way ANOVA with a follow-up Tukey multiple comparison test. *p<0.05; ***p<0.001 (TIFF 482 kb) [file 401_2018_1859_MOESM2_ESM.tiff]

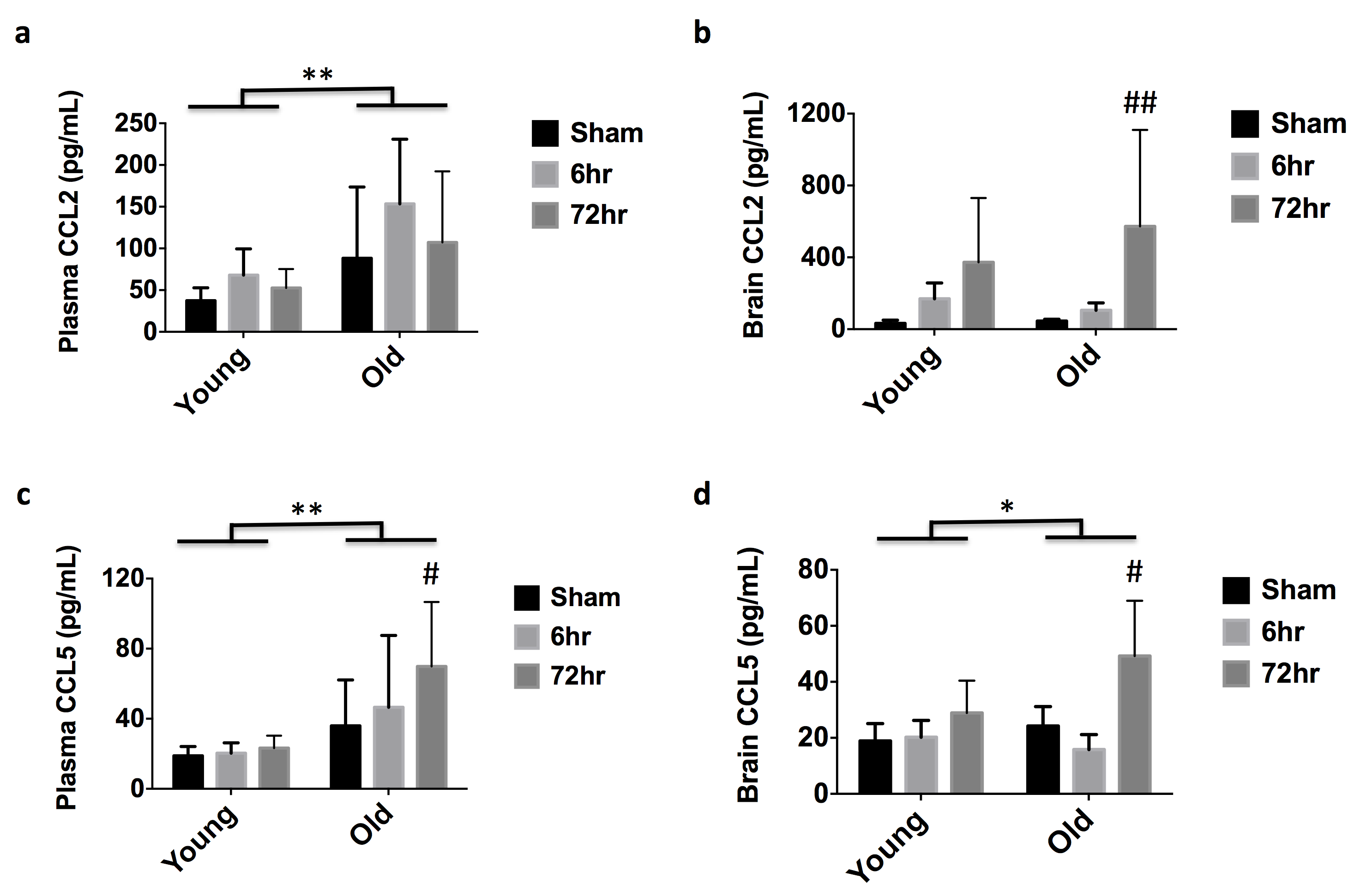

Supplement: Supplementary file 3 — Supplemental Figure 3. Effect of age on CCL2 and CCL5 concentrations after ischemic stroke. Plasma and brain concentrations of the chemokine CCL2 were elevated with age and after stroke in old mice compared to young mice (a and b, respectively; N=5-7/group). A significant effect of age (F(1, 30)=9.447, P=0.0045) and stroke (F(2, 32)=10.09, P=0.0004) was seen on CCL2 levels in the plasma and brain, respectively. CCL2 protein was significantly higher in the brain at 72 hrs after stroke relative to sham compared to young as determined by two-way ANOVA with post-hoc Tukey test for multiple comparisons. Plasma and brain concentrations of the chemokine RANTES/CCL5 were elevated with age and after stroke in old mice compared to young mice (a and b, respectively; N=5-7/group). CCL5 protein was significantly higher in old plasma and brain at 72 hrs after stroke compared to young as denoted by #. Error bars show mean SD. Abbreviation: CCL2 chemokine (C-C motif) ligand 2, SD standard deviation. **p<0.01 (TIFF 434 kb) [file 401_2018_1859_MOESM3_ESM.tiff]

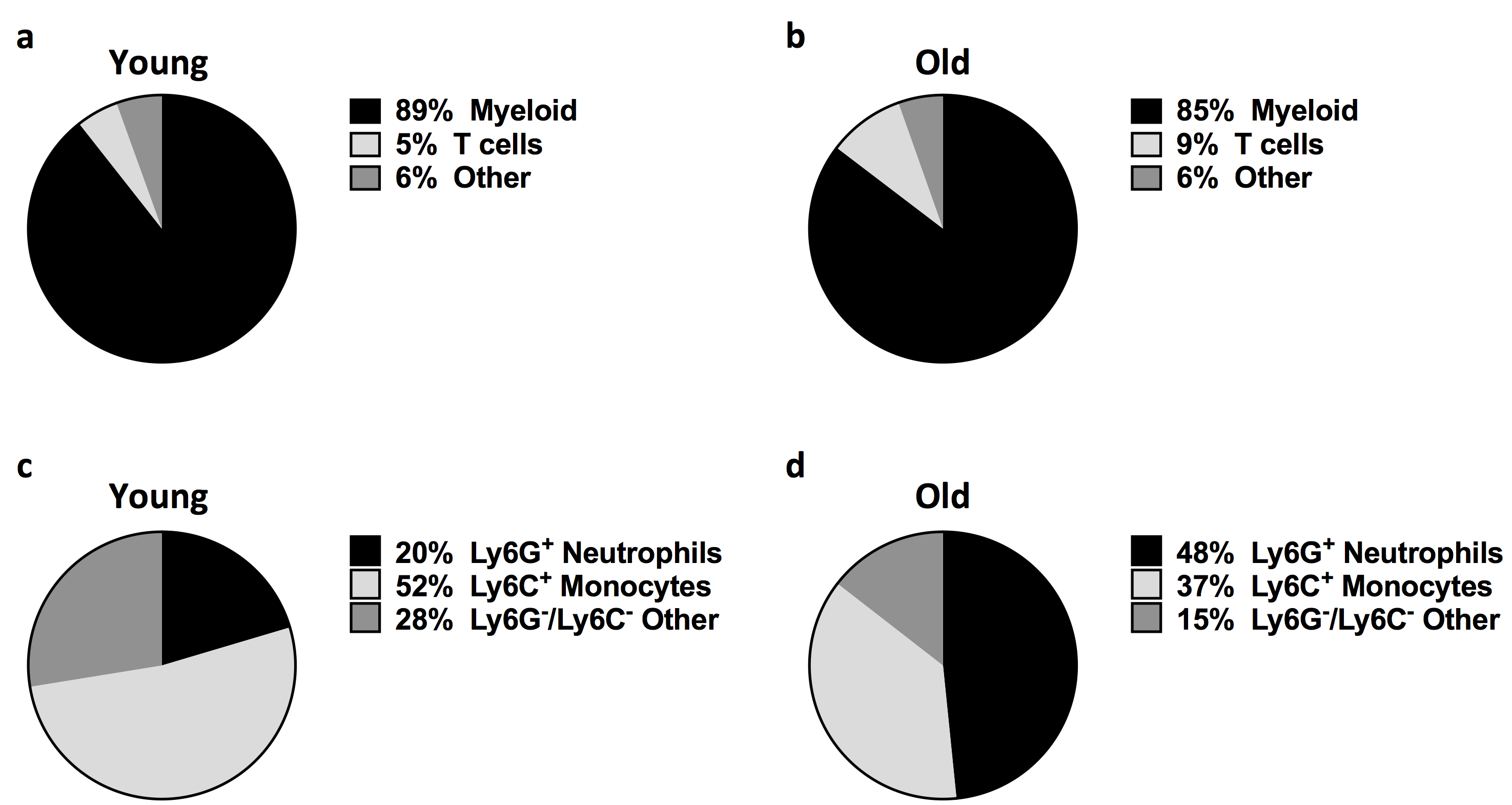

Supplement: Supplementary file 4 — Supplemental Figure 4. Age-related changes in brain-infiltrating leukocyte composition after stroke. Pie charts illustrate significant infiltration of cells of myeloid origin in both young (a) and aged (b) ischemic brains at 72 hrs in a cohort of injured animals. Compositional analysis of this bulk myeloid population reveal a substantial age-related bias in specific subsets found in young (c) and old (d) mice, including Ly6C+ monocytes and Ly6G+ neutrophils. (TIFF 331 kb) [file 401_2018_1859_MOESM4_ESM.tiff]

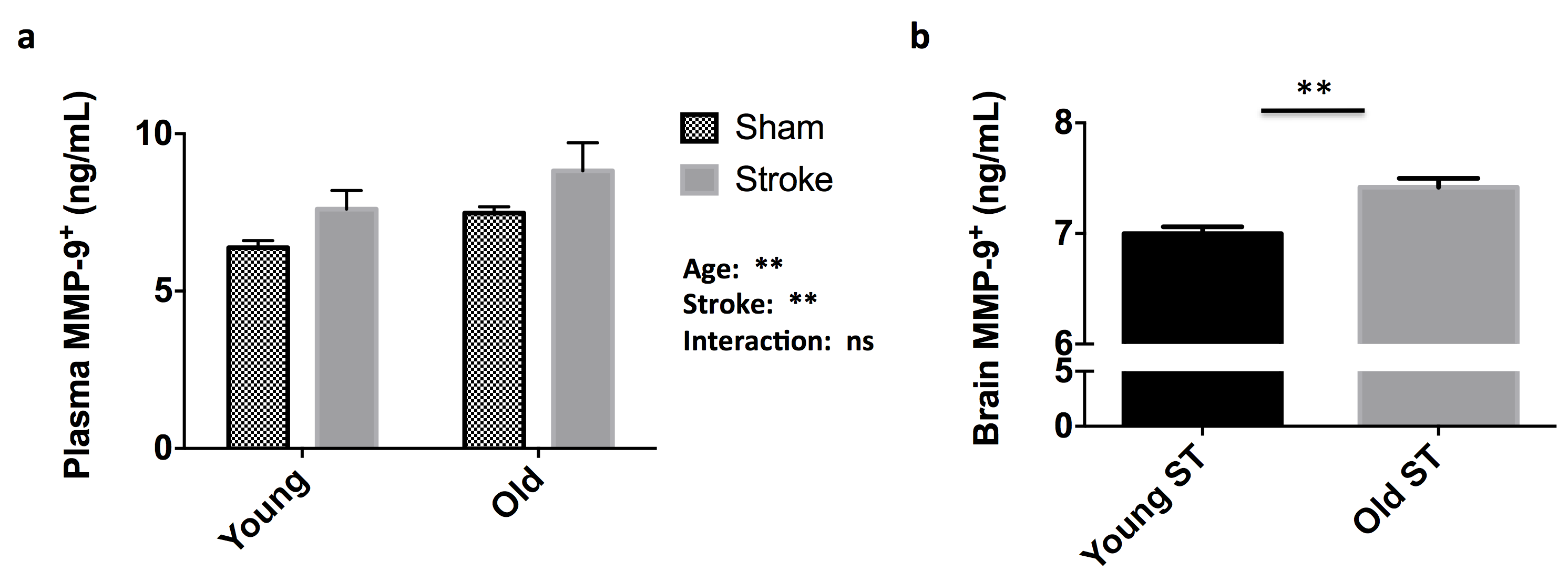

Supplement: Supplementary file 5 — Supplemental Figure 5. Plasma and brain MMP-9 concentrations in aging mice after ischemic stroke. Plasma concentrations of MMP-9 were measured in young and old mice at 72 hrs after stroke (a). Concentrations were analyzed by two-way ANOVA with post-hoc Tukey test for multiple comparisons (N=4/group). Brain concentrations of MMP-9 are shown for the ischemic brain and analyzed by unpaired, two-tailed Student’s t-test (b; N=4/group). Error bars show mean SD. Abbreviation: mL milliliter, MMP-9 matrix metalloproteinase-9, ng nanogram, SD standard deviation, ST stroke. *p<0.05; **p<0.01; ***p<0.001 (TIFF 260 kb) [file 401_2018_1859_MOESM5_ESM.tiff]

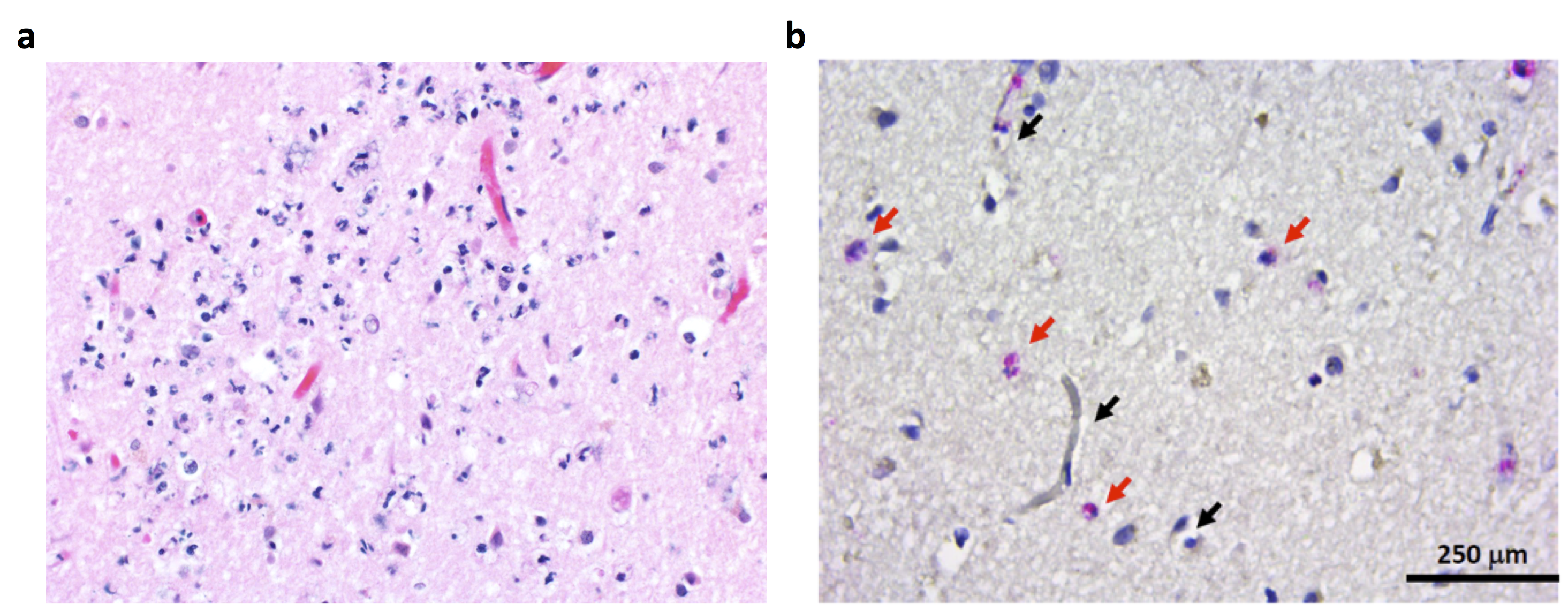

Supplement: Supplementary file 6 — Supplemental Figure 6. Histological assessment of neutrophil extravasation into the parenchyma after stroke in humans. Observational evidence for the transmigration of neutrophils into brain parenchyma was determined by H & E staining and immunohistochemistry of post-mortem human brain tissue from acute ischemic stroke subjects. Higher magnification of H & E staining shows congested capillaries (visible as bright red lines in the image) with a dense cluster of neutrophils in between the vessels (a). There are also a few residual dead-red ischemic neurons in the picture. Immunostaining for laminin alpha 5, a component of the endothelial basement membrane, shows that neutrophils are found in parenchymal areas outside of the basement membrane (b; 20X, scale bar = 250 μm). Red arrows indicate MPO-positive neutrophils and black arrows indicate endothelial basement membrane. Abbreviation: H & E hematoxylin and eosin. (TIFF 5141 kb) [file 401_2018_1859_MOESM6_ESM.tiff]
